# Therapeutic Applications and Mechanisms of Superoxide Dismutase (SOD) in Different Pathogenesis

**DOI:** 10.3390/biom15081130

**Published:** 2025-08-05

**Authors:** Shehwaz Anwar, Tarique Sarwar, Amjad Ali Khan, Arshad Husain Rahmani

**Affiliations:** 1 Department of Medical Laboratory Technology, Mohan Institute of Nursing and Paramedical Sciences, Bareilly 243302, India; shehwazanwar25@gmail.com; 2 Department of Medical Laboratories, College of Applied Medical Sciences, Qassim University, Buraydah 51452, Saudi Arabia; t.sarwar@qu.edu.sa; 3 Department of Basic Health Sciences, College of Applied Medical Sciences, Qassim University, Buraydah 51452, Saudi Arabia; akhan@qu.edu.sa

**Keywords:** reactive oxygen species, oxidative stress, antioxidant defenses, superoxide dismutase, therapeutic potential

## Abstract

An imbalance between the generation of reactive oxygen species (ROS) and antioxidant defenses is known as oxidative stress, and it is implicated in a number of diseases. The superoxide radical O^2–^ is produced by numerous biochemically relevant redox processes and is thought to play role in diseases and pathological processes, such as aging, cancer, membrane or DNA damage, etc.; SOD, or superoxide dismutase, is essential for reducing oxidative stress. As a result, the elimination of ROS by SOD may be a useful disease prevention tactic. There have been reports of protective effects against neurodegeneration, apoptosis, carcinogenesis, and radiation. Exogenous SODs’ low bioavailability has drawn criticism. However, this restriction might be removed, and interest in SOD’s medicinal qualities increased with advancements in its formulation. This review discusses the findings of human and animal studies that support the benefits of SOD enzyme regulation in reducing oxidative stress in various ways. Additionally, this review summarizes contemporary understandings of the biology of Cu/Zn superoxide dismutase 1 (SOD1) from SOD1 genetics and its therapeutic potential.

## 1. Introduction

**Figure 1:**
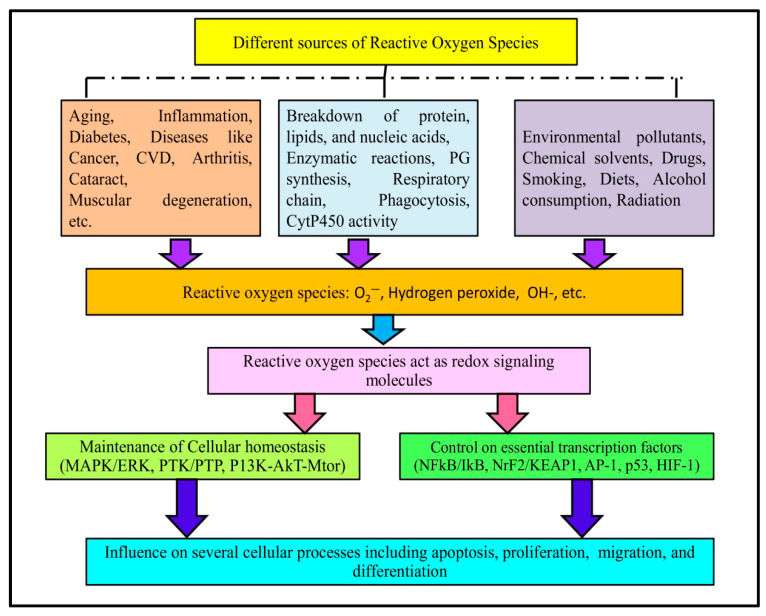
Examples of different metabolic, pathophysiological and environmental factors responsible for producing different ROS. These ROS can regulate different cellular signaling molecules and transcription factors, ultimately influencing several major cellular activities.

Reactive oxygen species (ROS) are very reactive oxygen-containing chemical entities that are constantly produced by the body as a result of cell metabolism (Figure 1). Oxidative stress is the outcome of increased ROS production and inadequate ROS sequestration. Proteins, nucleic acids, lipids, lipoproteins, carbohydrates, and connective tissue macromolecules can all be modified by oxidative stress, with varying susceptibility. Some may undergo reversible modifications, while others may experience oxidative damage depending on the intensity and duration of oxidative stress. Numerous severe diseases, including aging, inflammation, cancer, diabetes, cardiovascular disease, arthritis, cataracts, muscle degeneration, poor wound healing, etc., have been linked to the pathophysiology of oxidative stress and ROS [1,2]. ROS and reactive nitrogen species (RNS) are endogenous and highly reactive molecules and are produced during different enzymatic as well as non-enzymatic reactions in a biological system. Enzymatic reactions involving ROS production include prostaglandin synthesis, respiratory chain, phagocytosis, and cytochrome P450 system, etc. [3].

Moreover, both endogenous and exogenous sources contribute to the production of these species. In brief, endogenous sources include cancer, inflammation, excessive exercise, infection, immune cell activation, ischemia, aging, and mental stress. On the other hand, exposure to environmental pollutants, chemical solvents, certain drugs (cyclosporine, tacrolimus, gentamycin, and bleomycin), smoking and diet, alcohol consumption, and radiation are few examples of exogenous sources of free radicals [4]. ROS are implicated as a redox-signaling molecules in various cellular pathways involved in the maintenance of cellular homeostasis (MAPK/ERK, PTK/PTP, PI3K-AKT-mTOR), and controlling essential transcription factors (NFκB/IκB, Nrf2/KEAP1, AP-1, p53, HIF-1). As a result, ROS influence several cellular processes, such as apoptosis, proliferation, migration and differentiation [5,6]. In general, there is always redox balance maintained between the production and depletion of ROS. Antioxidants are molecules that prevent the oxidation of the substrate. However, in certain conditions, this redox balance becomes disrupted [7].

**Figure 2:**
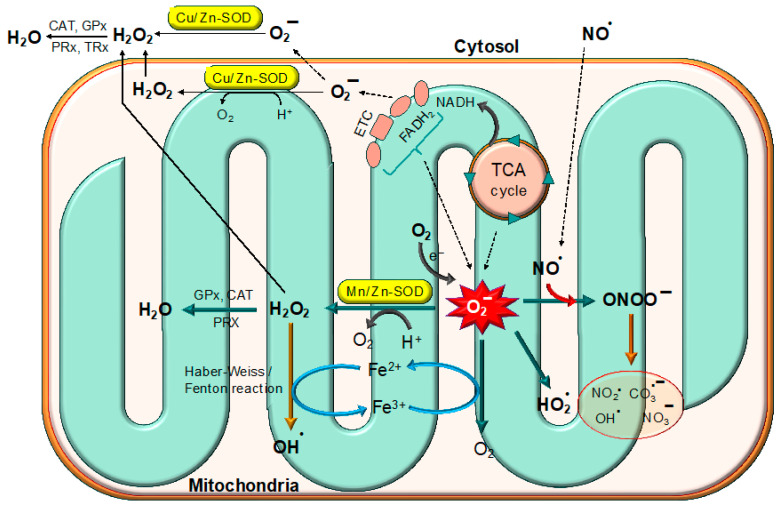
Superoxide free radicals are produced in mitochondria by the ETC chain and the TCA cycle. The superoxide radicals are detoxified by different superoxide dismutases (SODs) such as Mn/Zn-SOD and Cu/Zn-SOD.

Superoxide anion (O_2_^−^) is an important ROS and is formed by several enzymatic and non-enzymatic pathways (Figure 2). Like other ROS, O_2_^−^ causes oxidative damage to cellular biomolecules under specific conditions [8]. O_2_^−^ stimulates oxidation through various mechanisms such as the reduction of transition metals, the induction of metal release from proteins, forming conjugated acids in a pH-dependent manner and spontaneously dismutating itself into hydrogen peroxide [9]. According to previous reports, O_2_^−^ interacts with proteins, lipids, and nucleic acids more frequently than it does with low-molecular-weight antioxidants. Therefore, when compared to low-weight antioxidants, O_2_^−^ is a highly reactive oxygen species that is famous for its contribution in the pathogenesis of several diseases. The pathogenesis of O_2_^−^ depends on its concentration, location and cellular conditions [10].

There are several cellular antioxidants (both enzymatic and non-enzymatic) such as superoxide dismutase (SOD), catalase (CAT), glutathione peroxidase (GSH-Px), etc., that maintain a redox balance inside the cell. The excessive production and surplus accumulation of various ROS cause oxidative damage to different biomolecules, and are associated with various detrimental effects [5]. Free radicals have been implicated in the oxidative damaging of macromolecules such as proteins, lipids and nucleic acids as well as biochemical processes of biological system. Lipid peroxidation, structural changes and loss of enzymatic activity and the formation of DNA lesions are induced due to oxidative damage in these molecules. Oxidative damage might be involved in various human diseases that affect human health greatly. Oxidative stress has been recorded to be linked with multiple acute and chronic diseases including cancer, neurodegenerative disorder, cardiovascular diseases, diabetes, inflammation, etc., and acute pathologies [11,12].

This review aims to discuss SOD and its types, emphasizing bovine SOD, its structure and mechanism as well as its therapeutic uses for the management of several diseases. In addition, we highlighted the challenges and limitations associated with the therapeutic uses of SOD. Since liposomes are important delivery agents, we briefly discussed the utilization of liposomes as a carrier of SOD and the findings of studies documenting the significance of liposome–SOD conjugates as important therapeutic agents. At the end of this study, we highlighted carbon dots with SOD-like activities.

## 2. Superoxide Dismutase (SOD)

Superoxide dismutases (SODs) are very significant metalloenzymes and have been documented to be ubiquitously present across all domains of life. SODs play a crucial role in cellular defense mechanisms against oxidative stress. As the first line of defense against ROS-induced oxidative damage of biological system, SODs are crucial for maintaining oxidative homeostasis [13,14]. There are now three different forms of SOD found in mammalian cells including extracellular superoxide dismutase (EcSOD, SOD3), manganese superoxide dismutase (Mn-SOD, SOD2), and copper–zinc superoxide dismutase (Cu,Zn-SOD, SOD1) [15].

But according to the metal cofactors at their active sites, SODs can also be divided into four different types: Nickel SOD (Ni-SOD), manganese SOD (Mn-SOD), iron SOD (Fe-SOD) and copper–Zinc SOD (Cu,Zn-SOD) [14]. These SODs are found in different cell compartments and in all biological kingdoms in an uneven distribution. Cu/Zn-SOD is mostly found in the cytosol, mitochondria and chloroplasts, while Mn-SOD is primarily found in the peroxisomes and mitochondria. Conversely, mitochondria, chloroplasts, and peroxisomes are where Fe-SOD is primarily found. According to a comparison of the inferred amino acid sequences from Mn-SOD, Fe-SOD, and Cu,Zn-SODs, Mn-SOD and Fe-SOD are thought to be older SODs. The same ancestral enzyme is most likely the source of these enzymes. In contrast, Cu,Zn-SODs likely originated independently in eukaryotes and had no sequence resemblance to Mn-SODs and Fe-SODs [16].

The nuclear-encoded enzyme Mn-SOD moves into the matrix of the mitochondria, and it is a homotetramer with manganese as a cofactor and an active site. Manganese appears to be the ideal metal for preventing oxidative stress, because Mn-SOD’s quaternary structure appropriately preserves its catalytic and dismutase activity. From transcription and translation to posttranslational modifications, Mn-SOD expression and activity can be controlled on several levels. The primary transcription factors that control the expression of the Mn-SOD gene are NF-κB, specificity protein 1 (Sp1), activating protein-1 (Ap1), p53, and CCAAT binding protein (C/EBP), which either directly bind to particular DNA regions or interact with its partners [15].

The Mn-SOD and Fe-SOD groups are closely linked to one another, according to sequence and structural comparisons. According to their structural similarities, Mn-SOD and Fe-SOD seem to be different forms of the same enzyme [17]. Unlike the Greek crucial β-barrel of CuZnSOD, both have an α/β fold [18]. Usually, Mn-SOD and Fe-SOD are seen to be homodimers or homotetramers. There is a metal ion attached to every 200-residue monomer. These enzymes have specialized active sites for the superoxide anion and the corresponding metal ions. They have a conserved structure with a shell of residues enclosing a collection of metal-binding residues. The replacement of the equivalent metal ion in the native SOD reduces enzyme activity even though both enzymes can bind either Mn or Fe [19].

Because of their remarkable similarities, Fe-SODs and Mn-SODs are usually thought to have shared an ancestor. Changes in oxygen levels on early earth may have contributed to the distinct evolution of Fe-SODs and Mn-SODs. For instance, several archaea, such *Aeropyrum pernix* and *Pyrobaculum calidifontis*, have specialized Mn/Fe-SODs that are active with Fe but prefer to bind Mn, particularly in aerobic environments [20]. Lastly, Ni superoxide dismutase (Ni-SOD) has been found in marine species of *Synechococcus* and *Prochlorococcus* [21]. Ni-SOD is the only Ni enzyme that is involved in both nitrogen fixation and photosynthesis [22].

Cu,Zn-SOD is the primary copper-containing metalloenzyme in eukaryotes and it is enzymatic action was not identified until 1969, when McCord and Fridovich found its ability to dismutate superoxide anion [23]. It is extensively found throughout the cell membrane, nucleus, and cytoplasm [24]. It is a 32 kDa dimeric metalloprotein that is extremely soluble in its active state [25]. It is made up of two monomers, each of which has a single disulfide bridge. In Cu,Zn-SOD, one zinc ion, which mainly supports protein stability, and one copper ion, which serves as an enzyme catalyst, are bound by each monomer [26]. Cu,Zn-SODs have a copper atom acting as the catalytic cofactor and a zinc atom providing structure. While the copper atom is delivered by a copper chaperone via a translocation mechanism in a process that necessitates the development of a SOD-copper chaperone heterodimer, the zinc atom is most likely to be transferred to the enzyme in vivo by passive diffusion [27].

The Zn(II)-binding HVGD sequence (fragments 80–83) and the Cu(II)-binding HVH sequence (fragments 46–48) are the two binding sites of the Cu,Zn-SOD enzyme. The imidazolatee ring of His63 connects the two metal ions [27,28]. The quantity of copper binding at which the re-metalated derivatives exhibit SOD activity is proportional to the concentration of copper binding at the site of copper [16]. In 2021, ion Mobility-–Mass Spectrometry was used to examine the Cu,Zn-SOD Unfolding Pathway. First, elongation was shown when the holo-dimer was progressively denatured by applying various solution conditions. This was followed by dissociation into holo-, single-metal, and apo-monomeric SOD1 units. It was discovered that the loss of the metal cofactors further destabilized the protein monomers, leading to the development of apo-monomers that were incredibly stretched [26].

There are two types of Cu,Zn-SOD: extracellular Cu,Zn SOD (ecCu,Zn-SOD), which is encoded by SOD3, and intracellular Cu,Zn-SOD (icCu,Zn-SOD), which is represented by the SOD1 gene. The eukaryotic cytoplasm, as well as the mitochondria, peroxisome, and chloroplast matrix, contain the greatest distribution of icCu,Zn-SOD in living things [15]. According to research on their subcellular location in hepatocytes, Cu,Zn-SOD (SOD1), which contains copper and zinc, can be found in the intermembrane space between the mitochondria as well as in the cytoplasm, peroxisomes, lysosomes, and nuclei [29].

## 3. Bovine Cu,Zn-SODs

**Figure 3:**
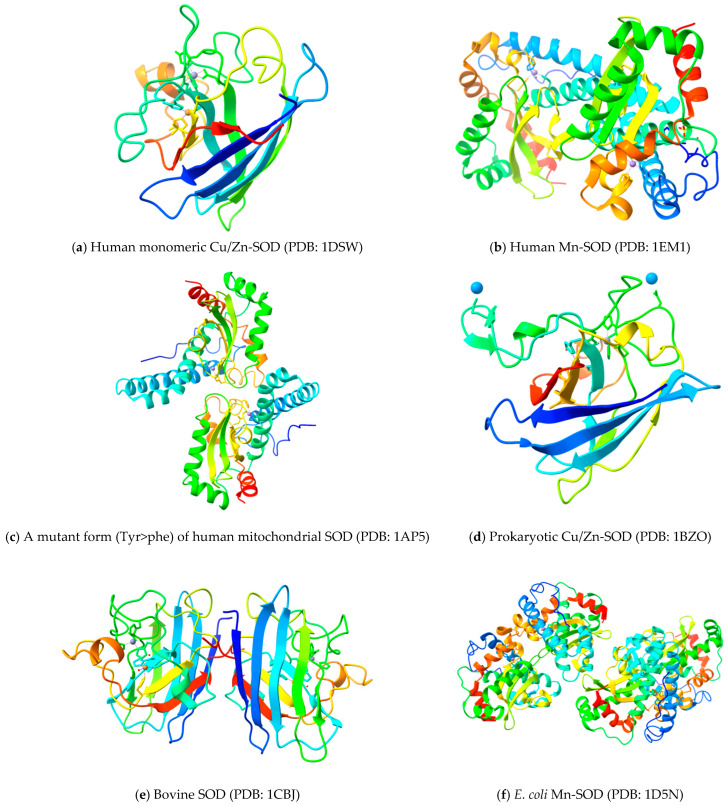
Three-dimensional structure of different SOD forms in different organisms with mentioned PDB codes.

Cu,Zn-SOD, the product of the SOD1 gene from bovine erythrocytes (BtCu,Zn-SOD, PDB code 2SOD), was first fully three-dimensionally modeled in 1982 [18]. Human Cu,Zn-SOD’s structure (HsCu,Zn-SOD, PDB code 1SOS) was solved in 1992 [30], demonstrating that the fold and domain organization of the enzyme were highly conserved in eukaryotes and later offering insights into amyotrophic lateral sclerosis (ALS) [31]. Numerous Cu,Zn-SOD structures from various eukaryotic species have since been identified. The structure of this eukaryotic enzyme is highly conserved across species [32]. These include the nuclear magnetic resonance (NMR) structures of the human Cu,Zn-SOD [33], and the X-ray crystal structures of the Cu,Zn-SODs from budding yeast [34,35,36], trematodes [37], frogs [38], spinach [39], and flowers of *P. atrosanguina* [40]. Human Cu,Zn-SOD has a high degree of sequence homology (83%) with the bovine Cu,Zn-SOD, along with similarities in three-dimensional structure, domain organization, amino acid sequence, protein folding, and catalytic properties [41,42]. The three-dimensional structures of different SOD forms in different organisms with the mentioned protein data bank (PDB) codes are provided in Figure 3.

Eukaryotic Cu,Zn-SODs are substantially conserved from the primary to quaternary structure, as demonstrated by the first BtCu,Zn-SOD and HsCu,Zn-SOD structures. Cu,Zn-SOD is a homodimer of 15.9 kDa that is stable. Hydrophobic interactions hold the dimerization in place, decreasing solvent accessibility and enhancing stability [29]. There are two functional areas in the SOD1 protein. The Cu,Zn binding region has 11–154 amino acids, and the amino binding region has 1–10 amino acids. Copper is found at active site of Cu,Zn-SOD [16].

Bovine Cu,Zn-SODis composed of two identical subunits and these subunits are oriented around an approximate two-fold symmetry axis at the dimeric interface [43]. The active site channel is made up of two additional significant external loops. Cu and Zn ions are present at the active site of the 32-kDa homodimeric metalloenzyme known as eukaryotic SOD1 in their active form [44]. In it, a β-barrel made up of eight antiparallel β-strands organized in a Greek key pattern makes up each subunit [45] with three external loops. These Greek key loops contain two conserved Leu residues, known as the cork residues, which fill the ends of the β-barrel. The core of this barrel is made up of densely packed hydrophobic residues, whereas the +3 β-strand Greek key connections (GK1 and GK2) are formed by loops β3/β4 and β6/β7 [18,46]. With three lengthy loops (loops IV, VI, and VII) and four conserved cysteine residues, the protomer of SOD1 forms an eight-stranded Greek-key βbarrel structure. Loop IV binds the Cu and Zn ions in the active site, and the highly conserved intramolecular disulfide bond between Cys57 in loop IV and Cys146 in the eighth β-strand (β8) stabilizes its shape [44].

Metal-free denatured monomer, which is the point of entry for filament formation in pathological conditions, was produced by the cleavage of the intramolecular disulfide bond and the loss of metal ions. Seldom does the SOD1 protein form oligomers in vitro without a reducing factor [44]. The β-barrel’s separation into two halves is one of its distinguishing features. On the outside of this barrel, the first half is present which has a normal, less twisted structure. The second half, on the other hand, is more twisted, less regular, and mostly located inside the subunit [45]. The walls of the active site are made up of two loops, the electrostatic loop and the zinc-binding site, as well as a section of the β-barrel [47]. Electrostatic interactions during substrate binding are facilitated by the presence of many charged residues in the electrostatic loop [48,49]. The third loop connects the two sides of the β-sheet. There is a small cavity about 10 Å below the protein’s surface that contains the active site. One copper (Cu) ion and one zinc (Zn) ion are found in each subunit of Cu,Zn-SOD [43]. Both Cu and Zn ions are important for preserving the structural stability of the enzyme [42,50,51]. The catalytic activity in converting superoxide radicals to hydrogen peroxide and dioxygen is attributed to the redox-active copper ion. Since the removal of the Zn^2^+ ion instantly inactivates SOD1, it is hypothesized that this ion aids in the development and durability of the native structure [50].

According to the enzyme’s structural analysis, the zinc (II) ion is bound by three histidine imidazole nitrogens and the aspartic acid’s carboxylate group, whereas the copper (II) ion is coordinated by four histidine imidazole nitrogens and an imidazolato bridge of His61 connects Cu(II) with Zn(II) [52,53]. The reduced Cu ion has three coordination sites and it adopts trigonal planar geometry. It is ligated by three histidine residues (His 44, His 46, and His 118) [54,55]. Three histidines (His 61, His 69, and His 78) and one aspartic acid residue (Asp 81) coordinate the Zn ion, giving it a warped tetrahedral geometry [45]. Each subunit contains a total of eight histidine residues [55]. NMR structural analysis demonstrates that the Cu ion is solvent-exposed, while the Zn ion is more deeply embedded within the protein structure. Additionally, bovine Cu,Zn-SOD possesses a disulfide bond [18] between a highly conserved pair of cysteine residues, specifically Cys 55 and Cys 144 [43]. The interaction of the active site metal ions and the conserved disulfide bond in each subunit is thought to contribute significantly to the stability of the enzyme [32]. Modifications to metal-binding regions could change the structure of SOD1 and encourage enzyme dysfunction. One of the mechanisms in AD has been suggested to be protein instability, which results in protein aggregation [56].

In brief, Cu,Zn-SOD exhibits highly conserved structural structure across eukaryotic species, including its β-barrel architecture, metal-binding sites, and disulfide bonds, which are essential for stability and catalytic function. The disruption of these structural elements can impair enzymatic activity and may contribute to disease-related protein aggregation.

## 4. Catalytic Mechanism of SOD

**Figure 4:**
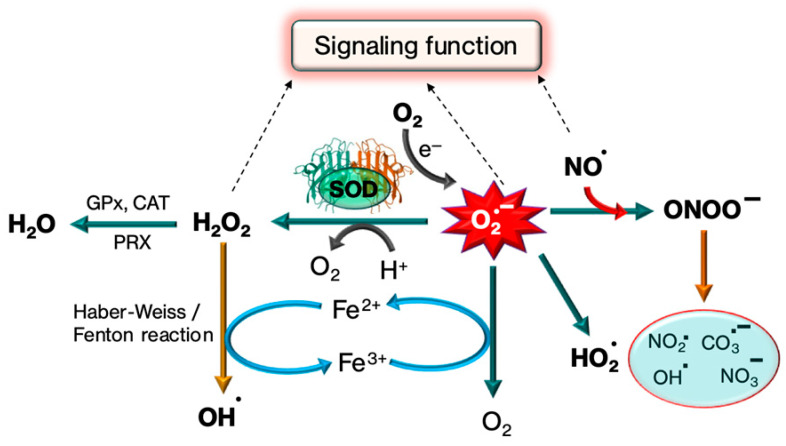
Mechanism of SOD in detoxifying the production of superoxide free radicals by the production of H_2_O_2_. Further, superoxide free radicals, H_2_O_2_ and NO free radicals perform some signaling function.

Through the alternating reduction and oxidation of the active-site copper, Cu,Zn-SOD catalyzes the extremely quick two-step dismutation of the harmful superoxide radical (O_2_^−^) to molecular oxygen and hydrogen peroxide [57]. The schematic representation of the dismutation of (O_2_^−^) by SOD is provided in Figure 4.

(1) $$\;{O_{2}}^{-}+{\mathrm{Cu}}^{2+},\;Zn-SOD\to O_{2}+{\mathrm{Cu}}^{+},\;Zn-SOD$$

(2) $$\;{O_{2}}^{-}+{\mathrm{Cu}}^{+},\;Zn-SOD+2H^{+}\to H_{2}O_{2}+{\mathrm{Cu}}^{2+},\;Zn-SOD$$

It involves two near diffusion-limited processes [32]. The oxidation of superoxide radicals to oxygen molecules and the creation of cuprous species Cu(I) in a trigonal planar configuration are the initial reactions that occur when superoxide radicals (O_2_^−^) bond to Cu(II) ions. This is followed by an inner-sphere electron transfer reaction. Partially protonated and prepared for reduction, Arg143 forms an anion-binding site to which a second equivalent of superoxide is bound. A proton from His63 and an electron from Cu(I) are donated to superoxide to produce hydrogen peroxide in the second reaction, which takes place in the outer sphere. At the same time, Cu(I) is oxidized back to cupric species Cu(II). One unpaired electron and nine d electrons, or d9, make up Cu(II) [58].

A charge gradient directs superoxide anion towards the active site since 89% of the exposed surface is negatively charged, whereas the active site and the channel leading to it are positively charged. About 11% of the accessible surface area is made up of 18 solvent-exposed residues that compose the active site channel’s surface. Glutamic acid residue located at position 133 (Glu133) and Lysine at position 136 (Lys136), two electrostatic loop residues, are very crucial for guiding the oncoming O_2_^−^ anion’s long-range approach. Additionally, arginine residue at position 143 (Arg143) controls the direction of O_2_^−^ in the active site channel and works with Thr137 to restrict the size of anions that approach the Cu center. Changes to these and other important charged residues can control the antioxidant activity of SOD1. Acetylation or succinylation of lysine residue located at position 122 (Lys122) within the electrostatic loop reduces its net charge from + 1 to 0 or −1, respectively, hindering the electrostatic guiding of anionic O_2_ towards the active site. Silent information regulator 5 (SIRT5) eliminates these alterations, indicating that SIRT1 and SIRT5 play comparable regulatory roles in preserving SOD1 catalytic activity [59].

In brief, Cu,Zn-SOD catalyzes the rapid dismutation of superoxide anion via redox cycling of copper at its active sites, guided by key charged residues. Structural elements, including electrostatic loops and post-translational modifications, critically regulate enzymatic activity.

## 5. Therapeutic Effects of SOD

**Figure 5:**
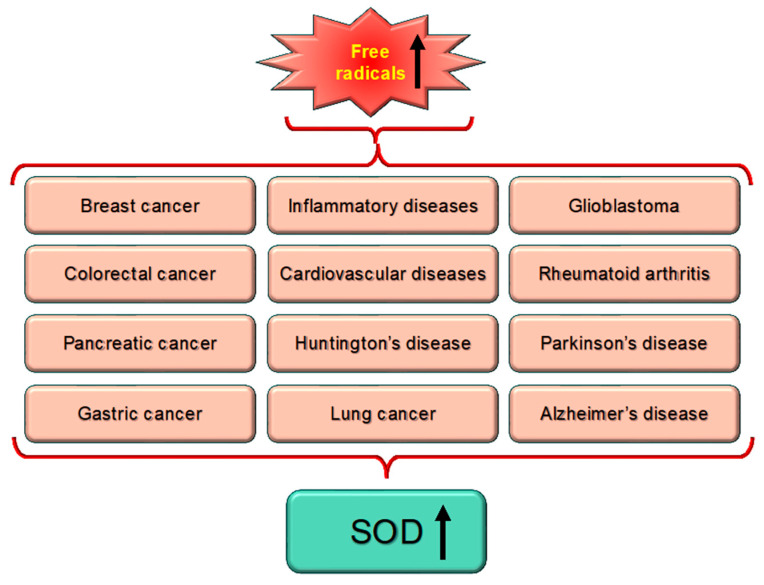
There exists a direct correlation between enhanced level of free radicals or oxidative stress that is linked with different diseases, which ultimately lead to enhanced level of SOD expression. Upward arrow indicated the elevated level.

Many diseases, including cancer, are linked to high levels of ROS or free radicals, which produce oxidative stress. Cu,Zn-SODs, antioxidant enzymes, are linked to a number of diseases because they are essential for detoxifying ROS (Figure 5).The most common pathological conditions such as inflammatory bowel disease, obesity and its consequences—diabetes and hypertension—and chronic obstructive pulmonary disease, have been linked to changes in Cu,Zn-SOD (SOD1 and SOD3) activity and its expression. Furthermore, the likelihood of acquiring a certain disease or its worsening has been linked to a number of SOD1 and SOD3 gene polymorphisms [60].

Numerous studies have demonstrated the diverse functions of mutant SOD1 using transgenic mice that express the protein. These include increased catalytic activity of peroxynitrite-mediated tyrosine nitration, easy release of reactive Cu ions, induction of apoptotic cell death, increased peroxidase activity, mitochondrial damage to release Ca^2+^, and formation of SOD1-containing aggregates in the cytoplasm. Downregulation of SOD1 in vitro and in vivo models has been associated with neuronal death [61], while overexpression of SOD1 in transgenic mice has been associated with protection of the cerebral tissue in several pathological conditions such as ischemia or Parkinson’s disease Lowered SOD activity has been linked to a high risk of oxidative stress, which can lead to a number of diseases, ranging including diabetes, heart failure, stroke, hypertension, high cholesterol, and atherosclerosis [14,62]. Consequently, it has been proposed that SOD supplementation’s antioxidant qualities can help with a range of pathophysiological issues, from immune system defense to aging prevention [62]. A summary of different studies emphasizing the implications of SOD in various diseases is provided in Table 1.

### 5.1. SOD and Cancer

As one of the leading causes of death across the world, cancer claims millions of lives each year [86]. Globally, cancer is the biggest cause of mortality. Approximately 7.6 million fatalities globally are attributed to it, and by 2030, that number is expected to rise to 13.1 million [87]. The development of cancer is caused by a combination of internal hereditary elements and several external influences. Understanding these elements can aid in reducing the risk of cancer development and carcinogenic exposures. Cancer cells exhibit high mutability due to internal mechanisms, like the accumulation of spontaneous mutations, as well as external influences, including environmental exposures and radiation [88]. ROS, which act as intermediates in both types of factors, can directly damage DNA. This damage includes DNA strand breaks, which disrupt the expression of essential genes such as proto-oncogenes, oncogenes, and genes involved in DNA repair, thus facilitating tumorigenesis [89,90].

Research by Zhang and colleagues demonstrates that lower SOD levels in patients with antineutrophil cytoplasmic antibody-associated vasculitis (AAV) reflect increased oxidative stress compared to healthy controls, highlighting the potential role of SOD as an important biomarker for rheumatologists. Inflammatory conditions activate neutrophils, which release large amounts of ROS. SOD not only decreases ROS levels but also helps regulate inflammation by modulating cellular signaling pathways [91]. Studies by Ueda and colleagues indicate that inflammation impacts SOD levels through transcriptional and proteolytic regulation, as both SOD mRNA and protein levels decrease during inflammation. In AAV patients, SOD levels negatively correlate with markers such as the erythrocyte sedimentation rate (ESR), C-reactive protein (CRP), and the Birmingham Vasculitis Activity Score (BVAS), suggesting that SOD decreases as inflammation intensifies [92].

Due to dysregulated metabolism, cancer cells often accumulate excessive ROS, which can lead to significant cellular damage and trigger apoptotic cell death. In response, these cells are under selective pressure to develop robust antioxidant defense mechanisms to counteract the cytotoxic effects of ROS. This is especially evident in advanced stages of cancer, where cells heavily rely on a highly active antioxidant system to support their rapid growth and survival. Specifically, two key enzymes—SOD1 and SOD2—protect cancer cells from oxidative damage. This dependence on antioxidant systems presents a promising therapeutic avenue, where targeting these enzymes could selectively inhibit cancer cell survival. Several compounds that target SODs have shown encouraging anticancer effects in preclinical studies, underscoring the potential of this strategy. Moving forward, enhancing the pharmacological properties of these compounds and gaining a deeper understanding of their mechanisms in various cancer types will be crucial. Additionally, combining SOD-targeting therapies with other modalities such as chemotherapy or radiation, which are known to elevate ROS levels, could further improve treatment efficacy and outcomes [93]. In summary, cancer progression is closely linked with oxidative stress, with SOD playing a key role in mitigating ROS-induced DNA damage and inflammation. In normal healthy cells, SODs protect against oxidative stress. However, cancer cells exploit the increased activity of SOD to survive oxidative stress, which makes SOD a promising target for anticancer therapies.

#### 5.1.1. SOD in Breast Cancer

In the context of breast cancer, alterations in the expression levels of SOD isoforms have been documented, with elevated SOD activity frequently associated with a worse prognosis. This increase in SOD activity may enable cancer cells to adapt to and survive in the oxidative stress conditions of the tumor microenvironment, promoting their proliferation [8]. Recent studies report altered expression of SOD isoforms, particularly in breast cancer, where elevated SOD levels support tumor survival under oxidative stress. The SOD levels may serve as diagnostic and prognostic markers.

The expression and specific activity of functional SODs seem to be increased by anastrozole treatment, suggesting that it may have a role in regulating antioxidant defenses in breast cancer [63]. In a study, in comparison to the control group, SOD activity increased after the second chemotherapy treatment cycle [94].

Modulating SOD levels or activity could improve treatment outcomes; for example, enhancing SOD activity may lead to increased oxidative stress, thereby inhibiting tumor growth. Conversely, reducing SOD activity could make cancer cells more vulnerable to chemotherapy and radiotherapy. Furthermore, measuring SOD levels may offer valuable insights for breast cancer diagnosis, prognosis, and monitoring treatment responses, paving the way for personalized therapeutic approaches. Current research is also exploring genetic factors, such as polymorphisms in the SOD genes, which could further elucidate the enzyme’s role in breast cancer susceptibility and management [95].

#### 5.1.2. SOD in Colorectal Cancer

Globally, colorectal cancer (CRC) is the second leading cause of cancer death and the third most frequent type of cancer. The epidemiology of colorectal cancer varies among populations, most likely as a result of exposure to environmental and lifestyle variables associated with the disease [96]. Colorectal cancer (CRC) predominantly affects individuals aged 30 years and above, with aging and prolonged exposure to free radicals contributing to heightened oxidative stress and increased cancer risk. Gender disparities are evident, with males comprising 73.5% of cases, a phenomenon linked to lifestyle factors like smoking and alcohol use, alongside the protective role of estrogen in females. Late-stage diagnosis (67.7% at stages III–IV) is prevalent due to socioeconomic barriers and the nonspecific nature of early symptoms, complicating treatment outcomes. Elevated SOD levels, averaging 2195.4 U/mL, are a hallmark of CRC, reflecting the body’s response to oxidative stress, with levels rising progressively with disease stage and malignancy. In this study, 4–5 mL of blood samples were collected from the peripheral vein of colorectal cancer patients. For the subsequent SOD assay, the blood serum was separated using a standard vacuum tube, aliquoted, and kept at −20 °C. Therefore, averaging 2195.4 U/mL refers to the SOD activity measured in blood serum [97]. Upregulating SOD3 reduces tumor development and liver metastases in colorectal cancer, indicating that SOD3 may have diagnostic and prognostic implications for the treatment of colorectal cancer [98].

Histopathological analyses show the highest SOD levels in moderately differentiated WHO Type I and III adenocarcinomas, highlighting its association with tumor progression. Changes in the expression levels of SOD isoforms have been identified in CRC, often linked to increased tumor aggressiveness and unfavorable prognosis. Modulating SOD activity offers promising therapeutic avenues; for instance, elevating SOD levels may amplify oxidative stress within cancer cells, making them more vulnerable to treatments such as chemotherapy. Moreover, SOD expression could function as a potential biomarker for diagnosing and predicting colorectal cancer outcomes, shedding light on tumor characteristics and patient treatment responses. Research into genetic variations in SOD genes also underscores their significance in CRC susceptibility and management, indicating that a deeper understanding of SOD’s roles may enhance therapeutic strategies [97]. A recent study shows that serum SOD activity and CRC risk are inversely correlated, especially in men and those with left-sided CRC or rectal cancer [67]. The rectal mucosa is protected by the safe and effective recombinant human SOD (rhSOD) enema, which dramatically lowers the frequency, intensity, and length of radiation-induced acute rectal injury (RARI) [99]. In conclusion, modulating SOD expression or activities may enhance treatment efficacy.

#### 5.1.3. SOD in Skin Cancer

Mn-SOD can alter a number of pathways that lead to the development of skin cancer. In an attempt to integrate antioxidant-based treatments into contemporary clinical practice, ongoing attempts are being made to create substances that efficiently trigger Mn-SOD [100]. Altered expression levels of SOD isoforms have been associated with skin tumor progression, where increased SOD activity may help cancer cells survive oxidative damage, promoting tumor growth. Additionally, SOD levels could serve as potential biomarkers for assessing skin cancer risk and prognosis, highlighting the enzyme’s importance in skin cancer prevention and treatment strategies. Understanding the mechanisms by which SOD influences skin carcinogenesis may open new avenues for targeted therapies aimed at enhancing antioxidant defenses in skin cells [101]. A study shows that Mn-SOD deficiency (Sod2+/− deletion) increases oxidative stress, AP-1 activation, and cell proliferation after DMBA/TPA treatment, whereas Mn-SOD overexpression inhibits the development of skin tumors by decreasing AP-1 activity [102]. In a DMBA/TPA two-stage skin carcinogenesis model, overexpression of the skin antioxidant enzymes Gsh-px or Gsh-Px + SOD increases cancer instead of decreasing it [103]. In contrast to nearby normal tissues, Cu,Zn-SOD activity was markedly decreased in a variety of malignant skin tumors, indicating a steady deterioration in antioxidant defense within tumor tissues despite individual diversity [104]. The chemoprotective efficacy of Boeravinone B (BB) against skin cancer produced by 7,12-dimethylbenz(a)anthracene (DMBA)/croton oil was examined in this experimental study, along with the potential mechanism. In DMBA/croton-induced skin cancer, BB therapy significantly (*p* < 0.001) decreased lipid peroxidation (LPO) and raised SOD, GSH, GSH-Px, and CAT levels [105]. These findings suggest that SOD has a dual role in skin cancer, with varying isoform expression impacting tumor development, antioxidant defense, and therapy responsiveness.

#### 5.1.4. SOD in Gliomas

SOD isoforms, especially SOD2 and SOD1, have been shown in prior studies to have dual functions in glioma and glioblastoma by shielding tumor cells from oxidative stress and enhancing resistance to treatment. Particularly when combined with mTORC1 inhibition techniques, their overexpression increases tumor aggressiveness and survival, making them both interesting therapeutic targets and possible prognostic indicators. Significant differences in enzyme activity among groups based on the molecular genetic profile and significant correlations between the peritumoral zone’s SOD activity and the number of tumor markers indicate the significance of evaluating SOD activity as a factor in the progression of gliomas [106].

MnSOD-transfected cell lines showed increased MnSOD immunoreactive protein, three to eight times the MnSOD enzymatic activity, and production of exogenous (plasmid) MnSOD mRNA. Compared to the parental and neo control cell lines, the MnSOD overexpressing cell lines grew in vitro at a greater serum concentration and grew tumors in nude mice significantly more slowly, indicating that they were less malignant. These results provide more evidence in favor of the theory that MnSOD functions as a tumor suppressor gene in a broad range of human malignancies [107]. The regulatory mechanism of the SP1/ZFPM2-AS1/miR-515-5p/SOD2 axis in gliomas was confirmed by a study, suggesting that ZFPM2-AS1 targeting could be a useful treatment strategy [108].

In glioblastoma (GB) cells, oxidative stress is triggered by both the tumor microenvironment and therapeutic treatments. SOD1 serves as a downstream target of the mammalian target of rapamycin complex 1 (mTORC1) [109], and mammalian target of rapamycin (mTOR) is thought to play a key role in promoting tumor cell survival and proliferation [110]. To explore this, SOD1 was inhibited in human primary GB cells through shRNA-mediated gene silencing, CRISPR/Cas9 knockout, and pharmacological inhibition. SOD1 activity was assessed using an SOD1/2 activity assay, and levels of ROS, cell death, and the NADPH/NADP+ ratio were measured under both normal and starvation conditions. In addition, mTORC1 activation in TSC2 knockdown cells (TSC2sh) was studied to examine the relationship between mTORC1 and SOD1. The results indicated that both genetic and pharmacological inhibition of SOD1 led to reduced SOD1 activity, elevated ROS levels, and increased susceptibility of GB cells to starvation- and hypoxia-induced cell death. This was associated with a decrease in the NADPH/NADP+ ratio. Interestingly, a combination therapy targeting both SOD1 and mTORC1 partially reversed the protective effect observed with mTORC1 inhibitor monotherapy. These findings suggest that SOD1 plays a pivotal role in helping GB cells adapt to stress in the tumor microenvironment through a mechanism that is dependent on mTORC1 signaling. Furthermore, the activation of SOD1 contributes to the resistance of GB cells to cell death induced by mTORC1 inhibitors, particularly under hypoxic conditions [111]. It was discovered that treatment with LCS-1 (an inhibitor of SOD) caused the simultaneous degradation of PARP and BRCA1, indicating that the failure of DNA damage repair may be linked to LCS-1-induced cell death. Together, these findings imply that SOD1 may be a target for glioma treatment and that the breakdown of PARP and BRCA1 may also be involved in the cell death brought on by SOD1 suppression [112]. According to a different study, SOD2 is essential for the tumor-initiating characteristics linked to temozolomide resistance. One possible therapeutic approach to improve the effects of chemotherapy is protein inhibition [113]. Gliomas exhibit elevated levels of ROS, which contribute to DNA damage and tumor growth. In gliomas, SOD2 is often overexpressed, enabling tumor cells to evade ROS-induced apoptosis and promoting their survival. However, this protective mechanism also contributes to treatment resistance, particularly against chemotherapy and radiation. Targeting SOD to increase ROS levels in tumor cells may enhance treatment effectiveness. Additionally, SOD2 expression is linked to tumor aggressiveness, making it a potential prognostic marker for gliomas. This dual role of SOD highlights its significance in both tumor survival and therapeutic targeting [114].

Thus, SOD isoforms, especially SOD2 and SOD1, have two functions in glioma and glioblastoma: they shield tumor cells from oxidative stress and help them become resistant to treatment. They are both promising therapeutic targets and possible prognostic markers because their overexpression increases tumor aggressiveness and survival, particularly when combined with mTORC1 inhibition techniques.

#### 5.1.5. SOD in Pancreatic Cancer

Pancreatic ductal adenocarcinomas (PDACs) are commonly linked to ROS. Therefore, an approach can be developed to create novel treatment options and find new biomarkers for PDAC diagnosis and prognosis by looking at the molecular and biological roles of SOD2. Nimbolide (NB) therapy of PDAC xenografts overexpressing SOD2 significantly reduced tumor development and metastasis [115].

Oxidative stress is a critical factor in pancreatic cancer progression. The role of enzymatic activity of SOD in pancreatic cancer is complex and context dependent. The excessive or dysregulated SOD activity has been implicated in fostering a tumor-supportive microenvironment and resistance to therapy. This dual role underscores the importance of carefully modulating SOD activity in therapeutic strategies against pancreatic cancer [15,16]. By up-regulating SOD2, pancreatic ductal adenocarcinoma (PPARγ) inhibited cell apoptosis, stabilized mitochondrial membrane potential (MMP), and reduced the production of mitochondrial reactive oxygen species (mitochondrial ROS). ATG4D-mediated mitophagy was then inhibited [116]. Together with the decreased Mn-SOD expression observed in pancreatic tumor formation, increased O_2_^−^ flow makes cells susceptible to treatments that aim to exploit the imbalance in pro-oxidants and antioxidants. Pro-apoptotic pathways can be activated by treatments intended to overwhelm these susceptible cells with an oxidative burst, which will ultimately cause cell death. It seems that the overexpression of the previously suppressed SODs is what creates the resistance that develops in surviving cells [117]. SOD activates the H_2_O_2_/ERK/NF-κB axis, which facilitates the epithelial–mesenchymal transition of pancreatic cancer cells [118]. In another study, inhibiting the H_2_O_2_/Akt/NF-κB axis with curcumin may be a promising therapeutic strategy for pancreatic cancer patients and curcumin might have potential to inhibit SOD-driven H_2_O_2_-induced pancreatic cancer metastases due to its capacity for the inhibition of PI3K/Akt/NF-κB signaling pathway [119].

The management of pancreatic ductal adenocarcinoma may benefit from altering SOD activity through inhibition, mimetics, or redox-targeted therapies.

#### 5.1.6. SOD in Oral Cancer

The concentration of SOD and the host environment at a particular location determine how it affects tumor cell development. A redox imbalance can contribute to the development of malignancies in oral cancer [120]. The markers of oxidative stress and antioxidant systems in the serum and saliva of patients with oral submucous fibrosis (OSF) and healthy controls are methodically examined in a meta-analysis. SOD levels were found to be considerably lower in OSF patients than in healthy controls [121]. The findings of a meta-analysis show the OSCC group’s serum levels of reduced glutathione (GSH), glutathione peroxidase (GPx), malondialdehyde (MDA), SOD, and MDA and GSH in saliva were dramatically altered in comparison to healthy controls. According to this study, a few biomarkers of oxidative stress might be useful in the early detection of OSCC [122].

A study investigated the comparative analysis of serum nitric oxide (NO) and SOD levels as potential therapeutic and prognostic biomarkers in OSMF and squamous cell carcinoma (SCC). A total of 87 participants were divided into three groups: Group I (OSMF), Group II (SCC), and Group III (healthy controls), with 29 individuals in each group. Venous blood samples were collected after an overnight fast to minimize dietary effects on serum beta-carotene, and standard protocols were followed for sample handling and processing. NO levels were measured using a modified copper-cadmium reduction method, while SOD levels were assessed using the Enzychrom™ SOD assay kit (BioAssay Systems, Hayward, CA, USA). The mean NO levels in Groups I, II, and III were 42.49, 50.08, and 32.81, respectively, while the mean SOD levels were 207.65, 196.93, and 226.57, respectively, with statistically significant differences (*p* < 0.001). The findings revealed elevated NO levels and reduced SOD levels in OSMF, with further pronounced changes in SCC, suggesting that these biomarkers could serve as valuable indicators for prognosis and therapeutic intervention [70]. According to a systematic review, the OSCC group’s mean SOD levels across a variety of biosamples were significantly lower than those of the normal controls [120]. SOD levels in a variety of human samples from oral submucous fibrosis (OSMF) patients were shown to be significantly reduced in metastatic research. Therefore, in order to support future therapy, more research is needed to quantify antioxidant status utilizing several biomarkers of different phases of the disease [123].

A systematic review and meta-analysis were conducted to look into the relationship between SOD levels and oral SCC (OSCC) and how it develops. While the OSCC tissue samples demonstrated a high immunoexpression of SOD in immunohistochemistry, tissue SOD values were considerably lower in the OSCC group than in the CG in all investigations that assessed tissue SOD levels by spectrophotometry [124].

A recent study assessed the levels of 8-OHDG, SOD, and GPx in the saliva of individuals with OSCC and OPMD who did not have diabetes mellitus. According to the study’s findings, patients with Type 2 Diabetes Mellitus who have OSCC and OPMD exhibit elevated oxidative stress and decreased antioxidant enzyme levels [125].

#### 5.1.7. SOD in Lung Cancer

In lung cancer, several SOD isoforms have different functions. Although SOD1 and SOD2 are frequently increased and linked to the development of tumors, SOD3 expression is significantly decreased, suggesting a compromised extracellular antioxidant defense. The most serious malignant tumor, lung cancer, requires the identification of novel molecular markers for diagnosis and prognosis. SOD3 is a secreted antioxidant enzyme that has the ability to regulate active oxygen in a microenvironment. SOD3 expression was low in lung cancer, and patients with high SOD3 expression had a lower survival rate [126].

SOD1 is a key antioxidant enzyme with known oncogenic roles in several human cancers. While SOD1 is frequently overexpressed in various malignancies, its clinical significance and functional roles in non-small cell lung cancer (NSCLC), especially regarding its epigenetic regulation in carcinogenesis and disease progression, remain underexplored. SOD1 expression was significantly elevated in NSCLC tissues and cell lines. Increased SOD1 levels were found to enhance NSCLC cell proliferation, migration, and invasion. Conversely, suppressing SOD1 expression led to G1-phase cell cycle arrest and induced apoptosis in NSCLC cells. Furthermore, miR-409-3p was identified as a negative regulator of SOD1, effectively counteracting its oncogenic effects. Bioinformatics analysis revealed that SET domain bifurcated histone lysine methyltransferase 1 (SETDB1) plays a role in the epigenetic modulation of both miR-409-3p and SOD1, forming a regulatory feed forward loop that influences NSCLC tumorigenesis and progression. These findings highlight the miR-409-3p/SOD1/SETDB1 axis as a critical mechanism in NSCLC and suggest that SOD1 could serve as a promising therapeutic target for treating this malignancy [127]. A study reported the modestly higher levels of Mn-SOD and CuZnSOD in lung carcinomas, with CuZnSOD activity almost doubling. On the other hand, there was a significant decrease in ECSOD expression, suggesting a loss of extracellular antioxidant defense, which could aid in the development of tumors [128]. When compared to patients with low serum SOD activity, this retrospective analysis showed that while high serum SOD activity may enhance post-radiotherapy quality of life in esophageal squamous cell carcinoma (ESCC) patients, it does not substantially increase patient survival [129]. A study concluded that serum SOD1 concentration was a better predictor than serum SOD2 concentration, however both SOD1 and SOD2 concentrations have been demonstrated to positively impact the risk of all-cause mortality in patients with lung cancer [130].

#### 5.1.8. SOD in Gastric Cancer

Gastric cancer (GC) was the fourth most common cause of cancer-related deaths globally in 2020 and the fifth most common type of cancer overall. One of the main risk factors for GC is *Helicobacter pylori* (*H. pylori*) infection, which the World Health Organization designated as a class I carcinogen in 1994. A study showed that SOD2 was elevated in gastric cancer (GC). Patients with GC who expressed more SOD2 had a worse overall survival rate. By transcriptionally stimulating the NF-κB signaling pathway, *H. pylori* infection increased the expression of SOD2. ROS and oxidative stress were elevated in response to *H. pylori* infection when SOD2 was knocked down [131]. A meta-analysis revealed that blood-based SOD activity was significantly lower in patients with gastric carcinoma compared to healthy controls. Subgroup analyses based on blood distribution and gender indicated that reduced SOD activity was particularly notable in erythrocytes and among male patients. These results suggest that SOD activity could serve as a supportive biochemical marker for the detection, monitoring, or prognosis of gastric cancer [132]. Similarly, significantly decreased SOD activity was reported in gastric cancer tissues compared to normal gastric tissues, further proposing reduced antioxidant activity as a potential prognostic marker for gastric cancer [133]. A substantial rise in SOD and CAT levels has been reported in the blood plasma of individuals with gastric cancer in stages III–IV. In group D, the rise in SOD and CAT levels in blood plasma was more pronounced than the rise in MDA content [134]. In addition to the development of neoplastic alterations in human gastrointestinal tissues, SOD1 and SOD2 also have a role in the progression of tumors in the following sequence: benign tumor—malignant tumor—metastasis [135]. In Cameroon, a cross-sectional investigation of dyspepsia patients revealed that those with *H. pylori* infection had noticeably greater levels of SOD activity and lipid peroxidation product. Additionally, these findings revealed that smokers only had increased lipid peroxidation when they had an H. pylori infection, and that SOD activity was considerably higher in persons over 50 [136].

In conclusion, SOD isoforms exhibit changed expression in gastric cancer and high levels are associated with activation brought on by *H. pylori* infection and a bad prognosis. Its potential as a predictive and diagnostic biomarker is highlighted by the fact that some studies show decreased systemic or tissue SOD levels, while others reveal increased SOD activity in advanced stages or in particular groups.

### 5.2. SOD in Inflammatory Diseases

Concurrent tissue damage and repair brought on by the inflammatory process is a sign of prolonged inflammation, sometimes commonly referred to as chronic inflammation. Additionally, it gradually alters the type of cells that are present in the vicinity of the inflammation. Inflammation is frequently linked to pain and is typified by a number of processes, such as altered membranes, increased vascular permeability, and protein denaturation [88]. People are burdened by chronic inflammatory systemic diseases (CIDs), such as multiple sclerosis, rheumatoid arthritis, and systemic lupus erythematosus, due to their high treatment and care expenses, increased mortality, and lifelong severe sickness [137]. Monocytes are bone marrow-derived circulating leukocytes of innate immunity that work with endothelial cells to coordinate tissue remodeling, angiogenesis, or inflammation in healthy or pathological settings. Chemokines and certain receptors draw monocytes to particular regions of arteries or tissues, where they undergo transdifferentiation into macrophages in response to infection or tissue damage. To cause vascular and tissue remodeling or to spread inflammatory reactions, adherent monocytes and infiltrating monocyte-derived macrophages locally produce a variety of cytokines, vasoactive agents, matrix metalloproteinases, and growth factors [138].

As the most prevalent leukocytes in the bloodstream, neutrophils are thought to represent the immune system’s first line of defense in the innate arm. Once pathogens are detected, they use phagocytosis, intracellular degradation, granule release, and neutrophil extracellular trap creation to catch and eliminate invasive germs. Additionally, neutrophils play a central and essential role in mediating inflammation [139]. Increased levels of O_2_^−^ are produced by stimulated neutrophils’ NADPH oxidase (NOX2), which serves as a precursor to hydrogen peroxide and other reactive oxygen species produced by their heme enzyme myeloperoxidase. O_2_^−^ is released by activated NOX2 on the internalized neutrophil membrane when neutrophils engulf bacteria in tiny vesicles called phagosomes. Myeloperoxidase uses hydrogen peroxide, which is produced when O_2_^−^ dismutates, to produce other oxidants, such as hypochlorous acid, a species that is extremely microbicidal [140].

SOD plays a crucial pathogenic role in inflammatory disorders. Although the relationship between the immune system and coagulation is not well understood, excessive inflammatory responses are becoming recognized as causes of coagulopathy during sepsis. By reducing endothelial dysfunction and minimizing the buildup of reactive oxygen species, extracellular SOD2 is required to trigger neutrophils’ antithrombotic action. In a lipopolysaccharide challenge murine model, antioxidants that intervene endothelial reactive oxygen species accumulation greatly improve disseminated intravascular coagulation and increase survival [140].

Acute respiratory distress syndrome (ARDS) is characterized by oxidative damage and dysregulated inflammation. A MnTE-2-PyP SOD mimic administered prior to treatment guards against acute lung damage, pulmonary neutrophilia, and platelet activation brought on by *Staphyloccocus aureus* [141]. Chronic and spontaneous inflammation in the gastrointestinal tract is a hallmark of inflammatory bowel disease (IBD), which has also been linked to elevated ROS levels. Through biomimetic mineralization, SOD was encapsulated inside a zeolitic imidazolate framework-zni (ZIF-zni) to create a nanocomposite known as SOD@ZIF-zni, which was then utilized as a formulation for the treatment of IBD. This SOD@ZIF-zni combination effectively reduced the level of ROS and pro-inflammatory cytokines in vitro and in mice model of dextran sulfate sodium-induced colitis. This study highlights therapeutic efficacy and biocompatibility of SOD@ZIF-zni for treating IBD [142]. SOD3 therapy prevented KLK-5-induced inflammatory cascades in SOD3 deficient mice. Similarly, SOD3 treatment also reduced KLK-5-induced inflammation in wild-type mice [143]. Severe morbidity and mortality are caused by acute pancreatitis (AP), which is a common inflammatory disease of the exocrine pancreas. Many reports indicate a link between this disease and cytoplasmic vacuolization, acinar cell death, edema formation, dysregulation of the production of digestive enzymes, and inflammatory cell infiltration into the pancreas. The acute inflammatory response is influenced by oxidative stress [144]. SOD1 may be protective against inflammation-induced oxidative damage in the group of acute pancreatic patients with the GC gentype [145]. Patients with severe acute pancreatitis who also have severe circulatory problems, renal failure, and a high death rate have far lower SOD activity. Acute inflammation is caused by some SOD gene polymorphisms, specifically acute destructive pancreatitis R213G. As a result, SOD is not only a crucial antioxidant enzyme but also a possible transcription factor that controls signaling pathway activity [146].

In short, SOD plays a critical role in mitigating oxidative stress and regulating immune responses in various inflammatory diseases. Its antioxidant function might be beneficial for ROS-mediated tissue damage. SOD-based therapies and mimetics show promise in conditions like IBD, ARDS, and acute pancreatitis

Inflammatory bowel disease and obesity are among the most prevalent chronic pathologies today, affecting a growing segment of the global population. IBD seems to occur in people who are genetically predisposed to immunological dysregulation and who have also probably been exposed to a number of environmental variables that increase their risk. As the prevalence of IBD rises along with obesity, especially in developing countries, there is increasing interest in the potential role obesity may have in both the pathophysiology and natural history of the disease. Obesity is on the rise in the IBD population with many estimates now citing that between 15% and 40% of these patients are obese, and both conditions have surged in parallel alongside modern lifestyle shifts such as urbanization and Westernized diets [147,148,149,150,151]. Diabetes and hypertension frequently co-occur with IBD and obesity due to shared risk factors, but current evidence report that these are comorbidities and are not consequences of IBD and obesity [152,153,154].

### 5.3. SOD in Cystic Fibrosis

A mutation in the cystic fibrosis transmembrane conductance regulator (CFTR) gene results in the malfunction of the CFTR protein, which in turn causes cystic fibrosis. Reduced transfer of chloride ions and a resulting dysregulation of the transport of mucus, the fluid lining the lung, pancreas, and other organs, occur when the protein is malfunctioning [155]. Compared to those with other diseases or healthy individuals, people with cystic fibrosis (CF) have higher levels of oxidative stress. This contributes significantly to the development of chronic lung injury by raising ROS and decreasing antioxidant molecules. Numerous clinical and preclinical studies indicate that the airways of a patient with CF present an intrinsically abnormal pro-inflammatory milieu due to elevated oxidative stress and abnormal lipid metabolism even before they become infected, despite the fact that it is known that recurrent infection–inflammation cycles in CF patients create a highly oxidative environment. This may have a direct connection to the deficit of the cystic fibrosis transmembrane conductance regulator (CFTR), which seems to cause a redox imbalance in extracellular fluids and epithelial cells [156]. The precise balance between oxidative agents and antioxidants, known as redox homeostasis, is essential for cellular processes and an organism’s overall health. Redox imbalance resulting in oxidative stress is recognized to occur when this redox homeostasis is disturbed by excessive ROS generation and decreased clearance, interfering with critical physiological and cellular signaling activities [157].

The enormous inflow of neutrophils into the airways and the presence of a malfunctioning CF transmembrane conductance regulator cause an imbalance in the processes of epithelial cells and extracellular fluids, which in turn causes an excess of reactive oxygen species and exacerbates oxidative stress. Both pulmonary and non-pulmonary CF symptoms contribute to oxidative stress in individuals. Massive neutrophil invasion of the airways, high ROS production, and granule release are the main ways that chronic respiratory tract infections cause an overreaction from the immune system. This results from neutrophil activity, which eliminates pathogens from the airways by inducing an oxidative burst [158].

RBC SOD activity was also investigated by Best et al. as a biological indicator of Cu status in CF patients. Together with the second Cu-dependent enzyme, plasma diamine oxidase, a decreased activity of this enzyme was observed in CF, although plasma ceruplasmin exhibited normal activity. The finding may be partially explained by the fact that copper deficit is known to accelerate the rates at which copper proteins degrade [159]. In 2024 a study was conducted on the effect of capsaicin on pulmonary fibrosis and bleomycin administered intratracheally caused oxidative damage, as seen by a marked rise in malondialdehyde (MDA) levels and protein carbonyl content. It also resulted in lower glutathione (GSH) levels, total antioxidant capabilities, and catalase and SOD activities compared to the control group [160]. Bleomycin was administered intratracheally on the first day and for the next twenty-one days in a different trial on pulmonary fibrosis. Compared to the sham groups, mice treated with the vehicle exhibited higher MDA levels and lower levels of SOD and GSH [161].

Assessing oxidation biomarkers and inflammatory levels in patients with CF and determining whether there is a correlation between these parameters and macrolide intake were the goals of a study. In addition to reduced levels of SOD, vitamin D, and vitamin A, the CF group showed significantly greater levels of interleukin-6 (IL-6), tumor necrosis alpha (TNF-α), reactive C protein (RCP), CAT activity, thiobarbituric acid reactive substances (TBARS), and isoprostanes [162]. Increased amounts of copper, iron, and zinc, decreased Cu/Zn- and Mn-SOD activity, and increased ROS and catalase activity were all observed in CF bronchial epithelial cells. Additionally, the investigation demonstrated the function of copper in CF-associated inflammatory processes and described the connection between inflammation and oxidative stress [163].

Hydrogen peroxide-induced apoptosis in basilar artery smooth muscle cells is inhibited by cystic fibrosis transmembrane conductance regulator (CFTR). According to this study, endogenous CFTR protein expression significantly decreased in tandem with H_2_O_2_-induced cell death. While adenovirus-mediated CFTR specific small interfering RNA (siRNA) reduced cell viability, the Bcl-2/Bax ratio, mitochondrial membrane potential, total glutathione levels, and the activities of SOD and catalase, it also increased H_2_O_2_-induced BASMC injury, mitochondrial cytochrome c release into cytoplasm, cleaved caspase-3 and -9 protein expression, and oxidized glutathione levels [164].

In one study, *Bacillus amyloliquefaciens* spores overexpressing SOD were introduced into a mouse model of bleomycin-induced lung fibrosis. The disease was greatly reduced by this treatment. Lung mRNA levels of connective tissue growth factor (CTGF), Col1a1, alpha-smooth muscle actin (α-SMA), transforming growth factor beta (TGF-β), TNF-α, and IL-6, as well as protein levels of all important markers of pulmonary fibrosis, such as TGF-β, Smad2/3, αSMA, and Col1a1 were shown to drop when mice were exposed to the spores [165].

In summary, cystic fibrosis is characterized by exacerbated oxidative stress and chronic inflammation due to CFTR malfunction. Reduced SOD activity contributes to this oxidative burden, and hence, restoring SOD levels may help alleviate inflammation and lung tissue damage in CF.

### 5.4. SOD in Cardiovascular Diseases

Heart failure (HF), arrhythmia, atherosclerosis, and stroke are among the disorders collectively referred to as cardiovascular disease (CVD). When the heart is unable to pump enough blood to support other organs in the body, two forms of heart failure can result. One is HF with decreased ejection fraction (HFrEF), or systolic HF caused by inadequate contractile performance. The other is HF with preserved ejection fraction (HFpEF), or diastolic HF caused by decreased cardiac filling in diastole [166]. A rise in the prevalence of age-related chronic diseases, particularly CVDs, such as hypertension, atherosclerosis, and heart failure, has coincided with the exponential growth of the world’s population over 60 in recent decades. The primary risk factor for these diseases is aging. The rise in oxidative stress, which damages cellular constituents like proteins, DNA, and lipids, at least partially explains this vulnerability to disease. Inflammation and oxidative stress are closely related processes that have a major role in the onset and advancement of cardiovascular disease. Blood pressure (BP) is regulated by the renin–angiotensin–aldosterone system, and its dysregulation can result in elevated ROS and inflammation in addition to BP abnormalities [167].

Heart failure is the most frequent consequence of acyanotic congenital heart disease (CHD), yet treatment and a conclusive diagnosis are still insufficient. The development of heart failure is frequently linked to the oxidative stress process. SOD and CAT levels in acyanotic CHD were significantly different in those with and without heart failure [168]. EC-SOD and DNA methyltransferase 1 (DNMT1) expression levels are measured using RT-PCR and Western blot analysis after ApoE-/-mice are given various diets for 15 weeks. The DNA methylation state may be changed by Hcy, and DNMT1 is a crucial enzyme in the methyl transfer process that can disrupt the EC-SOD DNA methylation status. This can result in a decrease in EC-SOD expression as well as an increase in oxidative stress and atherosclerosis [169]. This study investigated the association between the risk of ischemic stroke (IS) in the Chinese Han community of Dali City and single-nucleotide polymorphisms (SNPs) in SOD genes. TaqMan polymerase chain reaction was used to identify the SNPs rs17880487 and rs80265967 of the SOD1 gene, rs4880 and rs2842960 of the SOD2 gene, and rs2695232 and rs7655372 of the SOD3 gene. SOD3 levels of Rs7655372 were linked to a markedly elevated risk of IS. In the Dali region, the SOD3 gene rs7655372 locus polymorphism is a risk factor for IS [170].

The purpose of the study was to look at the relationship between the prevalence of CVD and the SOD1 polymorphism. In order to investigate the 50 bp insertion/deletion polymorphism (INS/DEL polymorphism) at the SOD1 promoter gene, a 6-year case–control follow-up research was established to genotype the 526 participants (311 controls and 215 cases) and examine their anthropometric traits and blood lipid profile. The results could have introduced the 50 bp INS/DEL polymorphism of SOD1 genotyping as a new and distinct diagnostic method for identifying high-risk cardiovascular diseases [171]. In patients with Type 2 Diabetes Mellitus (T2DM), atherothrombosis is the main cause of cardiovascular and cerebrovascular events, and platelet hyperactivation is essential for the development and progression of thrombotic problems that ensue from the disruption of atherosclerotic plaque. HPR+ patients in both T2DM and hypercholesterolemia (HC) have a lower activity of plasma extracellular SOD, and platelet response to collagen/epinephrine platelet function analyzer-100 (CEPI PFA-100) indicates that only SOD substantially predicted platelet reactivity. In conclusion, a suboptimal response to aspirin may be caused by the disruption of redox equilibrium linked to a decrease in SOD activity in both T2DM and HC [172]. The protection against ischemia and post-ischemic reperfusion damage of numerous organs and tissues, but especially of the myocardium, appears to be the most promising use of SOD in human medicine when considering the mortality and morbidity brought on by cardiovascular injury [173]. Since oxidative stress is a major cause of vascular problems, lowering cardiovascular risks in diabetics requires addressing oxidative stress and maintaining SOD function. Reduced SOD activity causes oxidative damage that interferes with NO signaling, which causes inflammation, vasoconstriction, and endothelial dysfunction [102,174]. A major source of disability and mortality in both industrialized and developing nations, stroke is a devastating cardiovascular disease. In the Dali region, the SOD3 gene rs7655372 locus polymorphism is a risk factor for ischemic stroke [170]. In order to determine the relationship between the quantity of coronary artery lesions and the degree of oxidative stress, a study was conducted to investigate the features of coronary artery lesions in patients with Type 2 Diabetes Mellitus (T2DM) who also had coronary heart disease (CHD). Compared to the CHD group, the SOD level was lower [175]. Angiogenesis was suppressed in endothelial cells by SOD1 inhibition, which also increased O_2_^−^ levels and decreased the phosphorylation of fibroblast growth factor-2 and VEGF-induced extracellular signal-regulated kinases. Vascular impairment in atherosclerotic and chronic pulmonary hypertension models in animals with SOD2 deficiency has been noted [176].

### 5.5. SOD in Aging

An increasing vulnerability of cells and tissues to harmful oxidative stress is one of the impairments caused by aging, which is marked by a steady reduction in physiological function [177]. Apoptosis and senescence can be triggered by a number of pathogenic alterations that oxidative stress induces in cells, such as mitochondrial failure, DNA damage, telomere shortening, lipid peroxidation, and protein oxidative modification. Numerous aging-related conditions, including cancer, ovarian disease, prostate disease, neurological disease, osteoarthritis, cardiovascular disease, and retinal disease, are also brought on by oxidative stress [178].

Atrophy, wrinkles, decreased tensile strength, and poor wound healing are all signs of aging skin. Dysfunctional fibroblasts also cause the loss of the collagen and elastic fiber network and structural integrity. Therefore, senescence has been modeled in vitro using dermal fibroblasts, not only for the dermis but also for other organs that are rich in connective tissue. Loss of collagen type I and type III, among other matrix constituents, dysregulated fibroblast matrix interactions, and reduced fibroblast interactions with organ parenchyma—primarily with muscle and organ-specific epithelial cells—are some of the alterations associated with aging skin. Both in vitro and in vivo studies have shown elevated ROS concentrations, fibroblasts that undergo a growth arrest, and morphological and functional alterations in human senescent skin [179]. ROS produced by the mitochondria have been directly studied in both mammalian and invertebrate model systems for their function in organismal aging. According to preliminary findings, oxidative damage—especially superoxide levels—do contribute to the shorter lifespans of invertebrates like *Caenorhabditis elegans* and *Drosophila melanogaster*. Although it is unclear how oxidative stress affects longevity in mammalian model systems, there is proof that antioxidant therapy protects against age-related dysfunction, including cognitive loss [180]. 

In vitro and in vivo tests were conducted utilizing fibroblast cell and D-galactose-induced aging-mouse models, respectively, to assess the inhibitory effects of highly stable SOD (hsSOD) against skin aging. Numerous studies, both in vitro and in vivo, have demonstrated the anti-aging effectiveness of hsSOD at the organ, tissue, cell, and molecular levels [181]. The age-related decline in SOD activity can lead to increased oxidative stress, contributing to the acceleration of aging-related diseases such as neurodegenerative disorders, cardiovascular diseases, and certain cancers. Ongoing research into SOD’s role in aging highlights its therapeutic potential, with investigations focused on SOD mimetics and supplements that enhance SOD activity in the body. By reducing oxidative damage, these interventions could offer promising strategies for improving health outcomes in aging populations and combating age-associated diseases [182]. A study examined the effects of taurine supplementation on oxidative stress biomarkers in women aged 55 to 70 years, based on the antioxidant properties of taurine, which can regulate oxidative stress in the aging process. Following taurine supplementation, an increase in the antioxidant marker SOD’s plasma concentration was noted [183]. Taurine is considered a cytoprotective substance because it can maintain a proper electron transport chain, preserve glutathione stores, boost antioxidant responses, improve membrane integrity, reduce inflammation, and stop calcium buildup [184]. In an effort to combat morphine-induced oxidative stress and hexabromocyclododecane (HBCD)-induced cytotoxicity, taurine treatment seems to enhance the activity of antioxidant enzymes (SOD, CAT, and GSH-Px) [185]. Taurine was able to prevent the decline in SOD activity and lessen the nitrotyrosine adducts that peroxinitrite caused. Tau can prevent CP-treated animals’ reduced activity of antioxidant enzymes (SOD, GPx, GR, and catalase) and non-enzymatic antioxidants (GSH) and preserve the best possible redox state under stress [186]. Tau may also maintain mitochondrial function, induce mitochondrial Mn-SOD, and control mitochondrial calcium homeostasis [187].

Extensive in vivo tests employing a D-galactose (D-gal) induced aging-mouse model confirmed that this SOD vehicle was highly effective in preventing skin aging and stimulating hair growth. Excellent hair follicle regeneration, higher melanin synthesis, reduced lipid oxidation, and improved skin tissue’s antioxidative capacity were all facilitated by SOD-polymeric needle (SOD-PMN) [188]. A SOD-containing dietary nutricosmetic called GliSODin^®^ Skin Nutrients Advanced Anti-Aging Formula (GAAF) (Isocell North America Inc., Toronto, Ontario, M5V 2B7, Canada) is made with additional nutraceuticals to support improvements in the skin’s hydration, elasticity, structural integrity, and photoaging brought on by oxidative stress. According to the study, the combination of generalized automatic anatomy finder (GAAF) and Tazarotene (TAZ) is safe and it offers substantial therapeutic benefits, including a relative improvement in fine wrinkles on the face, general photodamage, and skin elasticity and hydration [189]. Under typical circumstances, SOD-loaded exosomes (SOD@EXO) considerably increased the longevity of N2 wild-type *Caenorhabditis elegans* (*C. elegans*) in comparison to native SOD. Additionally, SOD@EXO increased resistance to oxidative stress and heat, resulting in a noteworthy survival ratio in these harsh environments. All things considered, the exosome-mediated delivery of SOD may lower ROS levels and postpone aging in the *C. elegans* model, offering possible future treatment approaches for disorders linked to ROS [190]. Treatment with dihydrotestosterone and recombinant EC-SOD increased collagen synthesis by activating TGFβ in human dermal fibroblasts. EC-SOD promotes collagen synthesis both in vitro and in vivo, hence preventing skin aging [191].

In summary, declining SOD activity contributing to tissue damage and dysfunction is a serious issue linked with aging. Enhancing SOD levels through supplements, mimetics, or advanced delivery systems has shown promise in reducing oxidative stress and delaying aging.

### 5.6. SOD and Rheumatoid Arthritis

Rheumatoid arthritis (RA) is a chronic inflammatory, autoimmune illness that mostly affects the synovial joints. As RA worsens, bone and cartilage degradation occurs. It raises mortality and morbidity ratios while decreasing patients’ functional ability. Numerous factors, both natural and genetic, interact to produce an inflammatory process that destroys the synovial membrane and causes an improper immune response adjustment [192]. Citrullination is the process by which major genetic factors (such as protein tyrosine phosphatase non-receptor type 22, interleukin-6 receptor, tumor necrosis factor receptor-associated factor-1, signal transducer and activator of transcription 4, peptidylarginine deiminase 4, CC chemokine ligand 21, DNA methylation changes, Fc gamma receptor, major histocompatibility complex regions encoding human leukocyte antigen (HLA) proteins) interact with environmental factors (such as air pollution, occupational dust, smoking, gut microbiota, unbalanced diet, etc.) to produce modified self-antigens [193]. Moreover, citrullinated proteins are no longer recognizable as self-structures by the immune system. The modified self-antigens are transported into the lymph node by antigen-presenting cells, which are activated to produce an immunological response. T cell activation takes place at this stage, which triggers costimulation to activate B cells [194].

Numerous studies have demonstrated how ROS contribute to the development of inflammation in chronic arthropathies like RA. Active polymorphonuclear cells (PMNs) and cell necrosis in an inflamed joint are the two primary ways that RA generates ROS. If these reactive species are not scavenged, lipid peroxidation takes place. More oxidation of polyunsaturated and unsaturated fats potentially results in cell membrane damage. In RA tissue and synovial fluids, lipoperoxidation products have been demonstrated to induce oxidative damage [192].

Because of its capacity to catalyze the dismutation of superoxide radicals implicated in the etiology of various inflammatory diseases, including rheumatoid arthritis, SOD is employed in antioxidant therapy. The main drawback of administering SOD in free form is that it does not accumulate well in inflammatory regions because of its short blood half-life and quick renal elimination [14]. In patients with rheumatoid arthritis and ankylosing spondylitis, the levels of SOD, MDA, erythrocyte sedimentation rate, and C-reactive protein (CRP) were measured. There was a substantial negative correlation (*p* < 0.001) between malondialdehyde and SOD [195].

The knee’s end-stage osteoarthritic synovium had substantially less SOD activity than the control synovium, which was unaffected by age. Although there was a little inverse relationship between aging and SOD activity, the SOD activity was substantially lower in the end-stage knee osteoarthritic cartilage than in the control. However, compared to control cartilage that was not impacted by aging, SOD activity in end-stage hip osteoarthritic cartilage was noticeably reduced [196]. A study was carried out to evaluate the correlation between the degree of lipid peroxidation as determined by blood and synovial fluid MDA levels, synovial fluid viscosity, disease activity, and the length of RA and the activities of antioxidant enzymes. The research groups exhibited considerably increased levels of Cu,Zn-SOD activity in blood, erythrocytes, and synovial fluid (SF). Human SF normally includes very little SOD, CAT, GSH-Px, and GST. As a result, ROS produced in the rheumatoid joint would not be effectively scavenged. Therefore, elevated lipid peroxidation in vivo is indicated by elevated MDA levels in SF from RA patients’ knee joints [197].

Thirty-six individuals with knee osteoarthritis, aged 50 to 70, were chosen for the study and split into two groups at random. Before and after the intervention, oxidative stress indicators like total antioxidant capacity (TAC), glutathione peroxidase (GSH-Px), SOD, and TBARS, as well as inflammatory markers like high sensitivity CRP (hs-CRP) and interleukin-6 (IL-6) were evaluated. According to the findings, burdock root tea considerably raised serum TAC levels and SOD activity, while dramatically lowering serum IL-6, hs-CRP, and MDA [198].

In summary, oxidative stress plays a critical role in RA pathogenesis and decreased SOD activity leads to joint damage and inflammation. Thus, enhancing SOD levels may be a promising therapeutic approach towards treating RA and associated arthritic conditions by preventing ROS-mediated tissue damage.

### 5.7. SOD and Neurodegenerative Diseases

Oxidative stress has been shown to be involved in the pathophysiology of several neurodegenarative diseases, which include a range of conditions that impact longevity and quality of life, such as Parkinson’s disease, Alzheimer’s disease, and motor neuron disease. Certain characteristics are shared by a number of the chronic neurodegenerative diseases of the central nervous system, including oxidative stress, inflammation, synapse dysfunction, protein misfolding, and impaired autophagia. Along with other forms of reactive oxygen species, neuroinflammation can also entail mast cell activation, which contributes to oxidative stress [199]. Cell membranes, proteins, lipoproteins, enzymes, hormones, and genetic material all undergo structural and functional alterations as a result of these extremely reactive substances. ROS primarily attack membranes in particular. Lipid peroxidation conversion products cause polyunsaturated fatty acids to break down and ultimately produce reactive aldehydes such as MDA and 4-hydroxynonenal (HNE). These substances alter the structure and functionality of DNA or protein molecules through reactions [200].

#### 5.7.1. SOD in Amyotrophic Lateral Sclerosis (ALS)

Both upper and lower motor neurons degenerate in amyotrophic lateral sclerosis (ALS) and it causes muscle weakness and ultimately paralysis. Whereas new imaging and neuropathological studies have shown that the non-motor neuraxis is involved in disease pathology, ALS was previously primarily classed within the neuromuscular domain [201]. Muscle weakness and atrophy develop gradually as a result of the loss of upper and lower motor neurons in the motor cortex, brain stem nuclei, and the anterior horn of the spinal cord. ALS frequently begins in a specific area and then spreads to other parts of the body, where respiratory muscle failure usually limits survival to two to five years after the disease first manifests. Extra-motor symptoms such behavioral abnormalities, executive dysfunction, and language issues might occur in as many as 50% of cases [202]. Kirby et al. originally reported the potential connection between SOD1 and Nrf2 in 2005. They discovered that the motor neurons of mice with the SOD1 (G93A) mutation had significantly lower Nrf2 mRNA levels and expression of Nrf2-dependent genes [203].

Mutations in the SOD1 gene are associated with the development of ALS, a progressive neurodegenerative disease that primarily impacts both upper and lower motor neurons. ALS exhibits significant variability in its clinical presentation, both between different families and among individuals within the same family, highlighting its heterogeneous nature [204]. After creating mice that expressed SOD1-G93A, Vargas et al. discovered that the astrocytes of these mice also showed elevated Nrf2 expression. Longer survival for the mice under study was the consequence of this increase in expression, which prevented degeneration in motor neurons where glutathione production was elevated [205]. In a cohort of 915 consecutively tested Polish ALS patients from a neuromuscular clinic, researchers identified causative variants in the SOD1 gene. They employed molecular modeling to study the structural effects of mutated SOD1 proteins, coupled with in silico analyses to evaluate the impact of these mutations on the clinical phenotype. Additionally, they conducted survival analysis to investigate associations between specific mutations and the risk of clinical outcomes. The study revealed 15 distinct SOD1 mutations, present in 21.1% of familial ALS cases and 2.3% of sporadic cases [206].

A mechanism involving SOD1 misfolding has attracted attention in recent years. The majority of human ALS cases with the SOD1 mutation and mice models of SOD1-ALS have strong SOD1 staining, suggesting of SOD1 aggregation, of hyaline inclusions. These hyaline inclusions/aggregates are considered the main neuropathological evidence for SOD1 misfolding in ALS, along with evidence from other neurological illnesses where protein aggregation and misfolding are believed to be involved. The pro-oxidation theory of SOD1-ALS is less strong now that it has been discovered that mutant SOD1 can produce an ALS-phenotype without copper loading into the active site [207].

In patients with ALS, mutations in SOD1 cause several changes in the structure and function of motor neurons [208]. As a result, the mutant enzymes cause misfolded protein chains, which in turn create tiny neurotoxic aggregates in the spinal cord’s glial cells’ (mostly astrocytes’) nuclei, causing neuron degeneration [209]. In patients with sALS, the SOD enzyme may undergo post-translational modification and hyperoxidation; as a result, changed SOD1 acquires hazardous qualities [210]. Furthermore, the SOD mutant exhibits decreased enzymatic activity, leading to an aberrant generation of ROS, which modifies cell function and induces necrosis and apoptosis [211].

In summary, mutations in the SOD1 gene contribute to ALS pathogenesis by promoting protein misfolding, aggregation, and oxidative stress. These alterations disrupt motor neuron function and survival, highlighting SOD1 as both biomarker and potential therapeutic target in ALS.

#### 5.7.2. SOD in Huntington’s Disease

The neurodegenerative condition known as Huntington’s disease (HD) is inherited and autosomal dominant. A dominantly inherited increase in the cytosine–adenine–guanine (CAG) trinucleotide repeat in the huntingtin gene on chromosome IV is the cause of the disease. Patients with HD typically have a 15–20-year disease course and exhibit a steady decrease in their physical and cognitive abilities [83]. In Huntington’s disease (HD), a neurodegenerative disorder marked by the accumulation of mutant huntingtin (HTT) protein, oxidative stress plays a significant role in neuronal injury. The presence of mutant HTT disrupts cellular homeostasis, resulting in the elevated production of ROS, which exacerbates neuronal damage [212].

SOD1 has been shown to play a protective role in HD. Decreased SOD activity in individuals with HD has been associated with an increased susceptibility of neurons to oxidative damage, which contributes to the ongoing neurodegeneration characteristic of the disorder. Research indicates that disturbances in antioxidant systems, including changes in SOD levels, worsen mitochondrial dysfunction and amplify cellular damage in HD. Consequently, strategies aimed at boosting SOD activity or regulating oxidative stress pathways are being explored as potential therapeutic options to slow the progression of neurodegeneration in HD [59,213].

A study was conducted for comparative analysis of SOD activity between acute pharmacological models and a transgenic mouse model of HD. Young transgenic mice showed higher levels of total SOD and Cu,Zn-SOD activity, but older (35 week) mice showed lower levels. A compensating strategy to shield cells from damage caused by free radicals is represented by increased enzyme activity; but, in older animals, the system is no longer adequate. Significant reductions in SOD activity were also noted following intrastriatal injections of quinolinic acid and 3-nitropropionic acid. It suggests that striatal oxidative damage happens in both kinds of HD models and is linked to changes in the antioxidant system within the cell [214]. While particulate SOD activity was reported to be unchanged in the HD parietal cortex and cerebellum, cytosolic SOD activity was shown to be somewhat decreased in these regions in a study on oxidative damage and metabolic dysfunction in HD. These findings provide more evidence that oxidative damage and metabolic abnormalities play a part in the pathophysiology of HD [215]. According to one study, HD patients had greater Cu,Zn-SOD activity [216].

Thus, altered activity of SOD in HD reflects its potential as a therapeutic target to mitigate oxidative damage in HD.

#### 5.7.3. SOD in Parkinson’s Disease

About 1% to 2% of adults over 65 suffer with Parkinson disease (PD), a crippling and incurable neurological disease. It is believed that oxidative damage is a major factor in Parkinson disease progression. In human SH-SY5Y neuroblastoma cells, a study showed the therapeutic potential of the SOD-mimetic chemical M40403 and the protective function of SOD enzymes against paraquat-induced toxicity. By functioning both cytosolically and mitochondrially, M40403 was able to make up for the loss of natural SOD enzymes in Drosophila [217]. Dopaminergic (DA) neurons in the substantia nigra pars compacta (SNpc) are lost in PD [218].

In PD, SOD is crucial for reducing oxidative stress, a key factor in the neurodegeneration associated with the condition. PD is marked by the gradual loss of dopaminergic neurons in the substantia nigra, an area of the brain essential for regulating movement. Oxidative stress plays a pivotal role in the damage to neurons that occurs in PD [219]. In PD, reduced function or dysregulation of SOD enzymes results in the buildup of ROS, which amplifies neuronal damage. This oxidative stress also promotes protein misfolding and fosters the accumulation of alpha-synuclein, a characteristic feature of PD pathology [220].

The largest risk factor for a number of neurodegenerative diseases, including idiopathic PD, is aging. Future treatments for a variety of diseases with oxidative stress-mediated pathology appear to be promising when it comes to SODs and SOD mimetics [213]. A study showed that PD patients had higher levels of red cell Cu/Zn-SOD activity, red cell copper and zinc, and plasma copper concentrations than older people without PD [221]. RBC-SOD/SOD1 and SOD1/RBC values were considerably decreased in PD patients, but ·OH and plasma-SOD values were significantly greater. RBC-SOD/SOD1 levels in PD patients were negatively connected with age and were much lower in older patients. PD patients who received pergolide therapy had significantly higher RBC-SOD/SOD1 values than those who did not receive either pergolide or bromocriptine medication. Therefore, pergolide may operate neuroprotectively by promoting SOD1 activity, and the greater ·OH level and reduced SOD1 activity may contribute to the start and progression of PD [222].

#### 5.7.4. Role of SOD in Alzheimer’s Disease

Despite decades of research and multiple clinical trials, the most common kind of dementia, Alzheimer’s disease (AD), still has no effective treatment. Although current treatment approaches have mostly focused on neuropathological characteristics such tau tangles and amyloid plaques, they have not been able to stop the disease’s progression, leaving patients with few alternatives [223].

In the central nervous system, misfolded proteins can cause oxidative damage, which can lead to neurodegenerative disorders in the mitochondria. Early mitochondrial dysfunction affects energy use in neurodegenerative diseases. Both tau and amyloid-ß issues impact mitochondria, resulting in mitochondrial dysfunction and, eventually, Alzheimer’s disease [224]. SOD1 in the cytosol and SOD2 in the mitochondria, play a key role in neutralizing superoxide radicals by converting them into less toxic molecules such as hydrogen peroxide and oxygen. In AD, excess ROS contributes to oxidative stress, which in turn promotes neuronal damage and accelerates the development of amyloid-beta plaques and tau tangles, the hallmark features of Alzheimer’s pathology [225,226]. By lowering lipid peroxidation and preserving hippocampal neurogenesis, SOD treatment in mice stopped cognitive impairment in stress-induced cells. Additionally, SOD reduced the amount of plaque in the brain while also lowering BACE1 expression [227]. The observed increase in the level of SOD following probiotic supplementation suggests a potential neuroprotective mechanism, as enhanced SOD activity may mitigate oxidative stress, thereby contributing to the preservation of cognitive function in AD disease [228].

Studies indicate that diminished SOD function, particularly within the mitochondria, results in increased oxidative stress, which further exacerbates synaptic dysfunction, neuronal death, and cognitive decline in AD. Strategies aimed at enhancing SOD activity or modulating oxidative stress pathways may provide promising therapeutic approaches to slow the progression of Alzheimer’s by reducing oxidative damage and safeguarding neuronal health [229,230].

### 5.8. SOD and Diabetes

Diabetes mellitus, which is characterized by high blood glucose levels, has emerged as a significant global health issue [231]. According to reports, glycation of biomolecules and diabetic consequences are largely caused by chronic hyperglycemia. Additionally, the buildup of AGEs (ultimate products of glycation) in vivo promotes the pathophysiology of diabetes by interacting with their receptors (RAGEs) and triggering the transcription of genes that regulate inflammation [232]. The most common pathological conditions in contemporary culture, such as inflammatory bowel disease, obesity and its consequences—diabetes and hypertension—and chronic obstructive pulmonary disease, have been linked to changes in SOD (SOD1 and SOD3) activity and its expression [60]. The current global epidemic of T2D has significantly increased, making up almost 90% of all cases of diabetes. Its mortality and worldwide economic burden are further increased by a variety of microvascular and macrovascular related disorders. Numerous functional aspects contribute to the development of diabetes and associated consequences. In particular, autoimmunity is crucial in causing pancreatic islet cells to become inflamed, which leads to β-cell failure in type 1 diabetes. Likewise, systemic low-grade inflammation serves as a shared mediator between the onset of type 2 diabetes and the development of micro/macrovascular problems [233]. Normal glucose homeostasis requires a healthy and functional quantity of pancreatic beta cells, and different degrees of beta-cell malfunction are associated with diabetes mellitus. Oxidative stress and a variety of pathogenic mechanisms lead to beta-cell malfunction [234].

Oxidative stress plays a crucial and fundamental role in the onset and progression of diabetes mellitus. Glycolytic, hexosamine, protein kinase C, polyol, and advanced glycation end-product (AGE) pathways are among the metabolic pathways where a number of molecular event cascades have been identified as pro-oxidative processes that are typically up-regulated in diabetics. A key factor in diabetes-related oxidative stress seems to be poly-ADP-ribose polymerase 1’s inhibition of glyceraldehyde-3-P dehydrogenase, which leads to the buildup of the enzyme substrate (glyceraldehyde-3-P) [235]. Patients with diabetes showed reduced SOD and GPx activity, with EBV(+) patients with DM2 showing the lowest values [236]. A study showed that diabetics had much lower levels of the antioxidant enzymes SOD and GPx. As the duration of diabetes increased, it was found that the levels of the antioxidant enzymes SOD and GPx decreased more. As a result, Type 2 diabetic patients are more vulnerable to oxidative stress-induced cellular damage [237].

Aspirin, a thromboxane synthesis suppressor that inhibits cyclooxygenase-1 (COX-1) irreversibly, is used to prevent cardiovascular diseases because platelet hyperactivation contributes to the known prothrombotic condition of metabolic diseases like Type 2 Diabetes Mellitus (T2DM) and familial hypercholesterolemia (HC). Similarly, a suboptimal response to aspirin may be caused by the disturbance of redox equilibrium linked to a decrease in SOD activity in T2DM and HC [172]. To evaluate the therapeutic potential of SOD in T2D, researchers administered oral SOD, liposome-encapsulated SOD (L-SOD), and SOD hydrolysate to diabetic model rats. Oxidative damage in the rats’ intestines was assessed through markers such as malondialdehyde levels, the GSSG/GSH ratio, and antioxidant enzyme activity. L-SOD showed the most significant improvement in repairing oxidative damage. Additionally, blood glucose levels and other metabolic markers correlated with oxidative damage and indicators of physical intestinal damage, including colon density, histological staining (H&E), and immunohistochemical analysis of tight junction proteins like occludin and ZO-1. The study also measured systemic inflammatory markers such as lipopolysaccharide and related cytokines. Among the SOD formulations, L-SOD proved to be the most effective, followed by regular SOD and SOD hydrolysate. These findings suggest that oral administration of SOD, particularly L-SOD, can reduce blood glucose levels by targeting intestinal oxidative stress and systemic inflammation in diabetic rats [238].

85 patients with T2DM were randomized to receive combination of SOD, alpha lipoic acid, acetyl l-carnitine, and vitamin B12 (B12) in a prospective, double-blind, placebo-controlled research. With the exception of CARTs and MNSIE, all peripheral neuropathy indicators, including SNAP and SNCV, pain, and Quality of Life perception, improved when the four components were combined into a single tablet and taken for a full year by patients with DMT2 [239]. Maternal diabetes causes autism-like behavior by suppressing SOD2 and causing prolonged oxidative stress due to hyperglycemia [240]. 

73 individuals with T2DM and Diabetic Neuropathy (DN) were randomly assigned to receive a single tablet containing ten components, including, palmitoylethanolamide, SOD (70 UI), alpha lipoic acid, and vitamins, etc. The combination of the ten components in a single pill for six months at a daily dosage of two tablets considerably increases B12 levels, pain, and vibration perception threshold [241].

In summary, oxidative stress contributes significantly in the onset and progression of diabetes. Additionally, diabetes mellitus is linked with reduced SOD activity, β-cell dysfunction, inflammation, and other complications. Numerous reports are available indicating decreased SOD levels in diabetic patients. Therefore, oral or combined SOD-based treatments can be beneficial for improving glucose control, neuropathic symptoms, and antioxidant defense.

### 5.9. SOD in Pneumonia and COVID-19

The most serious kind of acute lower respiratory tract infection that affects the lungs’ pulmonary parenchyma is pneumonia. It is a common illness that has a high risk of infection as well as substantial morbidity and mortality. The sixth most common cause of mortality is pneumonia. Endogenous sources of infections include, but are not limited to, nasal carriers, sinusitis, oropharynx, stomach or tracheal colonization, and hematogenous transmission. The body reacts by starting an inflammatory assault when germs enter the lower respiratory tract and lung parenchyma at the alveolar level. The main element of the pulmonary defense system is alveolar macrophages, which are immune cells that neutralize and eradicate bacterial growth. But as soon as a disease gets past the body’s defenses, it spreads [242]. The findings of a study imply that the host’s immune defenses may be somewhat lowered in impaired controls due to a lower activity concentration of SOD [243]. In one study, individuals with community-acquired pneumonia (CAP) had their platelet counts, SOD activity, and Cu and Zn concentrations in platelet-rich plasma assessed both at admission and at discharge. Compared to control persons, CAP patients had considerably reduced levels of Cu and all platelet SOD activity [244]. Another study found that lecithinized-SOD therapy was safe and increased serum levels of surfactant protein-A and lactate dehydrogenase in individuals with severe respiratory dysfunction and advanced idiopathic interstitial pneumonias [245].

According to immunohistochemistry, fibrotic regions and fibroblastic foci in usual interstitial pneumonia (UIP) lungs were remarkable for lacking extracellular SOD (ECSOD). ECSOD immunoreactivity was considerably reduced in fibrotic versus non-fibrotic regions of the sick lung, according to Western blotting. Interstitial mast cells were the only cell type to exhibit strong ECSOD positivity in UIP. ECSOD is generally quite low in fibrotic parts of UIP, which is consistent with a number of other antioxidant enzymes. This could potentially raise the oxidative burden in this disease [246]. Mice expressing the R213G EC-SOD variation were reported to be shielded from the extrapulmonary spread of germs even though their lung bacterial load was identical to that of wild type controls [247].

A potentially fatal virus, coronavirus disease-2019 (COVID-19) is brought on by the severe acute respiratory syndrome coronavirus 2 (SARS-CoV-2). With further development in the lower respiratory tract and gastrointestinal mucosa, the COVID-19 infection mainly targets the respiratory system through the mucosal epithelium of the upper respiratory tract (nasal cavity and pharynx). There have also been reports of non-respiratory symptoms in COVID patients, such as acute liver and heart attacks, diarrhea, and renal failure [248]. In COVID-19, SOD significantly decreased in activity, with severe patients showing lower activity than mild to moderate patients and the control group. Notably, the SOD activity was lowest in severe patients who passed away. Significant relationships between SOD and indications of coagulation malfunction, inflammation, organ damage, nutrition, and lymphocyte counts were found by correlation analysis [249]. 271 patients with moderate COVID-19 (132 males and 139 women) participated in a study. Using spectrophotometry, the amount of SOD in erythrocytes was determined. SOD activity was higher in women in the early reproductive age group than in those in the 36–45, 46–60, and 61–90 age groups. Women between the ages of 18 and 35 have higher SOD activity than men in this age range, according to the sex differences. When selecting treatment strategies for patients with moderate COVID-19, these findings should be considered [250].

The top five genes in the network analysis were CAT, NFE2L2, SOD1, SOD2, and CYBB. While the GSH level dropped in COVID-19 patient PBMCs, the expression of these genes and intracellular ROS/O_2_^−^ levels rose. In comparison to the control group, COVID-19 patients’ nasopharyngeal (NP) samples showed reduced expression of high-ranked genes. The total oxidant status (TOS) level and the activity of the extracellular enzymes SOD and CAT were elevated in COVID-19 patient plasma samples. Additionally, the best performance was demonstrated by the 3-marker panel and the 2-marker panel of CAT and TOS [251].

Because of its important function in the body’s reaction to infections, oxidative stress may have a major part in the pathogenesis of COVID-19. A significant elevation of cell death triggered by oxidative stress has been linked to viral pathogenesis and a malfunctioning redox balance. A total of108 COVID-19 patients and 28 controls had their blood samples taken, and oxidative stress-related metabolites were measured. While total antioxidant capacity (ABTS and FRAP) was reduced in COVID-19 patients, antioxidant enzymes (SOD and CAT) and oxidative cell damage (carbonyl and lipid peroxidation (LPO)) were significantly higher in these individuals [252].

By regulating oxidative stress and immunological responses, SOD plays a critical role in respiratory infections. SOD levels are frequently changed in COVID-19 and pneumonia, usually falling in severe cases, and they are correlated with organ damage, inflammation, and the severity of the illness. These results demonstrate the potential of SOD as a therapeutic target and biomarker for respiratory disorders.

## 6. Challenges and Constraints of Superoxide Dismutase in Therapeutic Applications

One of the primary limitations of using SODs as therapeutic agents is their poor bioavailability. Natural SODs, particularly when administered intravenously or orally, are prone to rapid degradation by proteases and immune clearance. This significantly reduces their effective concentration at the target site [58]. SODs have a relatively short half-life in the bloodstream, which limits their sustained therapeutic effect. Frequent administration would be necessary, which is not ideal for chronic conditions requiring long-term treatment [253]. SODs are large proteins, and their entry into cells is limited, which makes it difficult to target intracellular sites of oxidative damage. Effective delivery systems are required to ensure that SOD reaches specific cells and tissues, particularly in conditions like neurodegenerative diseases, ischemia–reperfusion injury, or cancer [16]. For neurological disorders such as glioma or stroke, crossing the blood–brain barrier is a significant challenge. SODs administered systemically may not effectively reach the brain without advanced delivery mechanisms, such as nanoparticle carriers or conjugation with targeting molecules [254]. SODs neutralize superoxide radicals indiscriminately, which can sometimes be problematic. While reducing ROS is beneficial for preventing oxidative damage, ROS also play essential roles in cellular signaling, immune responses, and apoptosis. Non-selective ROS scavenging by SOD may interfere with these physiological processes, potentially leading to unintended consequences [8].

Without precise targeting, SODs may act on both diseased and healthy tissues, disrupting normal cellular functions. This lack of specificity can cause imbalances in oxidative signaling pathways, which could negatively affect therapeutic outcomes [4].

Being foreign proteins, therapeutic SODs can trigger immune responses, leading to the formation of neutralizing antibodies or allergic reactions. This immunogenicity can reduce the effectiveness of treatment over time or cause adverse effects. Some patients may experience allergic reactions to therapeutic SOD formulations, limiting their use in sensitive populations [255]. The large-scale production of recombinant SODs can be expensive and technically challenging, given the need for proper folding, glycosylation, and stability of the enzyme. Producing SOD in a form that retains its functional activity after administration requires sophisticated biotechnological processes. Due to these challenges, the cost of producing and administering SOD therapeutics remains high, making it less accessible for widespread clinical use, particularly in resource-limited settings [256].

Despite promising preclinical studies, clinical trials of SOD therapies have often shown mixed or modest results. For example, trials for conditions such as ischemia–reperfusion injury, stroke, or neurodegenerative diseases have not consistently demonstrated significant benefits, largely due to the aforementioned challenges in delivery, bioavailability, and specificity [257]. Developing SODs or their mimetics as therapeutics also faces regulatory challenges due to the complexity of demonstrating consistent clinical efficacy and safety in large patient populations [258].

In summary, despite the therapeutic promise of SOD, its clinical application is hindered by poor bioavailability, limited tissue targeting, potential immunogenicity, and high production costs. Overcoming these issues through advanced delivery systems and careful targeting is essential for translating SOD-therapies into effective clinical treatments.

## 7. Pathologies Caused by Overexpression of SOD

While excessive oxidative stress is harmful, reducing ROS levels too much can create another imbalance, known as reductive stress. ROS are necessary for many cellular signaling pathways, and excessive scavenging by SODs could inhibit these processes, potentially leading to adverse effects. In some cases, overexpression of SODs has been linked to accelerated aging and other pathologies [259]. SODs are primarily responsible for neutralizing the O_2_^−^ that is created in the cytosol, mitochondria, and endoplasmic reticulum of cells in all aerobic species. However, because the dissociation of O_2_^°−^ produces H_2_O_2_, which is harmful to cells, the SOD may potentially have a pro-oxidant effect. The existence of additional antioxidant systems, like CAT and GSH-Px enzymes, is required to eliminate this hazardous H_2_O_2_ [260]. Numerous diseases, including Down syndrome (DS) and Alzheimer’s disease, have also been linked to the overexpression of Cu,Zn-SOD in a number of tissues. It has been proposed that the toxicity seen in cells overexpressing SOD is caused by elevated H_2_O_2_ concentrations and consequently enhanced hydroxyl radical oxidative damage [261]. One of the best-documented cases of a human condition aetiologically linked to a redox imbalance is DS, which has long been linked to overexpression of the trisomic chromosome 21-encoded enzyme Cu,Zn-SOD-1. It has been documented that oxidative stress plays a role in the transcriptional control of genes at other chromosomes as well as in genes other than those at chromosome 21 [262]. In DS patients with translocations between chromosomes 14–21, 21–21, and 10–21, the levels of Cu,Zn-SOD, CAT, GPx GR, and MDA were comparable to those of age-matched people with normal trisomy. Oxidative stress was shown to be reduced in patients with higher proportions of the normal cell line [263]. According to De la Torre and colleagues), DS patients with full trisomy 21 had higher erythrocyte SOD activity than karyotyped individuals (by 42% and 28%, respectively). The authors also noted translocations, mosaicism, and normal SOD activity in the population with partial trisomy 21 [264]. The myogenic function of human skeletal muscle-derived stem/progenitor cells is positively correlated with both transient and stable overexpression of extracellular SOD [265].

## 8. Approaches for Increasing the Therapeutic Efficacy of SOD

To overcome major limitations of native SOD including short half-life, poor bioavailability, and limited tissue targeting, various delivery approaches have been developed. Chemical modifications like PFGylation, lipidation, and ligand attachment have been shown to increase circulation time, reduce immunogenicity, and improve selective uptake of SOD. Encapsulation of SOD in carriers, such as liposomes, micelles, nanoparticles, and microparticles protects from degradation and enables controlled release and improved pharmacokineics. Additionally, oral formulations are also being explored to improve SOD absorption. For example, SOD mimetics have been designed and have been reported to overcome several limitations of native SOD [62]. The creation of novel SOD analogs and mimetics with better pharmacokinetic characteristics and increased therapeutic efficacy is another viable strategy for improving the clinical translation of SOD-based medicines [266]. SOD conjugates, SOD loaded into particulate carriers (micelles, liposomes, nanoparticles, and microparticles), and the most promising and appropriate formulations for oral delivery are used for increasing the therapeutic efficacy of SOD [62]. It has been discovered that a variety of polymeric nanoparticles, whether synthetic or natural, are useful for loading or encapsulating various natural bioactive substances to improve their bioavailability and therapeutic effectiveness.

One of the earliest methods for enhancing organ selectivity, membrane permeability, immunogenicity, pharmacokinetics, and protein and enzyme stability is ligand modification. Enhanced delivery systems, such as nanoparticle carriers and gene therapy, are being developed to overcome challenges related to poor bioavailability, stability, and specificity [266].

In an investigation, SOD and cationic functionalized chitosan were chemically attached to create the novel nanoparticle-like conjugate O-HTCC-SOD, which has shown greater promise than SOD in treating diseases linked to ROS. O-HTCC-SOD was first evaluated for its impact on rat chondrocyte exposure to monoiodoacetate (MIA), taking into account the role that ROS play in the pathophysiology of osteoarthritis. The focused intracellular ROS clearance capabilities and enhanced pharmacokinetic characteristics of the nanoparticle-like combination O-HTCC-SOD led to its exceptional efficacy [267]. In order to improve the stability and activity of SOD in a study, chitosan-coated alginate gel particles (CS/SA) and chitosan-coated alginate-shellac gel particles (CS/SA/Lac) were developed as a possible oral administration strategy. According to the results, it considerably increased SOD’s stability in stomach circumstances. SOD encapsulated in CS/SA was fully liberated in the activated intestinal fluid at optimal formulation [99]. In another study, targeting FSH peptide was reported to enhance Sertoli cell uptake of NPs. After six hours of H_2_O_2_-induced oxidative stress, FSH-conjugated SOD-NPs dramatically protected Sertoli cells, resulting in 100% survival as compared to unconjugated SOD-NPs (45%) or SOD in solution [268]. With a rat focal cerebral ischemia–reperfusion injury model, Reddy and Labhasetwer evaluated the effectiveness of SOD-NPs, which are biodegradable poly(D,L-lactideco-glycolide) nanoparticles encapsulated with SOD. The majority of critical neurological functions were later restored in animals treated with SOD-NPs, which also showed higher survival than those treated with saline control. For stroke patients, SOD-NPs may be a useful therapeutic alternative when used in combination with a thrombolytic medication [269]. Cell-free hemoglobins have been extensively studied as artificial oxygen carriers for use in blood transfusions for hemorrhagic shock, anemia, ischemic heart diseases, etc. Following conjugation, it was discovered that the enzymatic activities of the SOD and CAT in the conjugates (Hb–SOD–CAT) retained more than 70% and 90% of their initial bioactivity, respectively [270].

## 9. Liposomes: SOD Carrier

Drug delivery is the procedure of giving an animal or human a pharmacological ingredient that has a therapeutic effect. There are numerous opportunities for the creation of medications based on nanoparticles to identify, treat, and cure challenging conditions. By adjusting their size, shape, surface modification, surface features, and materials used, a variety of nanomaterials can be made into intelligent systems with discrete medical and imaging agents [271]. By boosting drug concentration, residence time in target cells, and reducing side effects, drug delivery systems (DDSs) have the potential to improve a medicine’s therapeutic index. Through improved drug pharmacokinetics and biodistribution, as well as serving as drug reservoirs, DDSs improve the pharmacological characteristics of free medicines and mask their negative qualities by delivering the potentially active drug to the site of action via a nano-vehicle. Depending on their intended use, these nanoparticles (NPs) typically ranged in size from a few nanometers to several hundred nanometers [272].

Liposomes are the most commonly used nanocarriers for a range of hydrophobic and hydrophilic chemicals that may be physiologically active due to their superior biocompatibility, biodegradability, and low immunogenicity. Furthermore, liposomes have demonstrated enhanced drug dissolution rate, controlled drug delivery, and surface modification capabilities. Based on how they form, liposomes have evolved from conventional and immune liposomes to stimuli-responsive and actively targeted liposomes [271]. Additionally, liposomes demonstrated improved drug solubility and controlled distribution, as well as the ability to modify their surface for targeted, sustained, and protracted release. Liposomes can be thought of as having changed from traditional, long-circulating, immune-system-targeted liposomes to stimuli-responsive, actively targeted liposomes based on their composition [272].

The antioxidant activity and intracellular delivery of SOD are hindered by its incapacity to penetrate cellular membranes. SOD entrapment in liposomes is one method for promoting intracellular transport of macromolecules. By adding pH-sensitive liposomes containing 1,2-dioleoyl-sn-glycero-3-phosphoethanolamine (DOPE) and 1-oleoyl-2-oleoyl-sn-glycero-3-succinate (DOSG) to cultivated fetal rat lung distal epithelial (FRLE) cells, SOD was delivered to lung cells [273]. In vitro reactive oxygen species toxicity and acute liver damage caused by lipopolysaccharide in D-galactosamine-sensitized mice were found to be prevented by polycationic liposome-mediated EC-SOD gene delivery [274]. The idea that a deficiency of SOD plays a role in the retinal vaso-attenuation observed when the animals are exposed to hyperoxic conditions was tested using a newborn rat model of retinopathy of prematurity. The successful delivery of SOD to the retina by long-circulating liposomes raises the possibility that restoring and/or enhancing endogenous antioxidants in oxygen-damaged retinal tissue could be a useful therapeutic approach [275].

In mice undergoing osteoarthritis (OA) surgery, intra-articular injections of SOD-NPs was reported to effectively inhibit the onset of OA and stopped its progression. As a result, this research shows that SOD-NPs have therapeutic potential and that OA treatment may benefit greatly by targeting the synovium [276]. It has been shown that liposome-embedded SOD reduces oxidative stress and intestinal barrier dysfunction in animals with DSS-induced ulcerative colitis [277]. In beagles, local administration of liposome-encapsulated catalase and SOD were documented to reduce periodontal inflammation [278]. The ability of variously charged liposomes to deliver Cu,Zn-SOD to human lung epithelial cells, A2182, and their potential for protecting cells from oxidative agents were investigated. Cellular glutathione levels were constant after pre-treating cells with liposome-encapsulated Cu,Zn-SOD, indicating protection against oxidative stress [279]. In order to alter the pharmacokinetics and biodistribution of SOD and boost its therapeutic activity, a study investigating liposomal administration was carried out. The findings suggest that SOD can be targeted to arthritic areas using tiny poly(ethyleneglycol)-liposomes following subcutaneous injection [280]. In contrast to intravenously injected catalase and superoxide dismutase, which had circulation half-lives of 23 and 6 min, respectively, liposome-entrapped catalase and superoxide dismutase activity had a half-life of 2.5 and 4 h [281]. Manganese superoxide dismutase-plasmid/liposomes delivered by inhalation shield the murine lung from radiation damage [282].

The most appealing formulation for in vivo testing seemed to be SOD-containing PEG-liposomes made using the dehydration–rehydration technique. In comparison to “free” SOD, both small PEG-liposomes and SA-liposomes had superior therapeutic effectiveness, with PEG-liposomes eliciting more potent anti-inflammatory effects than SA-liposomes [283]. Fasudil and SOD’s biological half-lives were considerably prolonged by CAR-liposomes [284].

In summary, liposomes serve as efficient carrier for SOD, enhancing its stability, cellular uptake, and therapeutic half- life, while minimizing toxicity. Their adaptability for targeted, sustained delivery makes them promising approaches for treating oxidative stress-related diseases. Continued advancements in liposomal formulations may further improve the clinical utility of SOD-based therapies.

## 10. Carbon Dots: SOD-like Activity

Numerous nanomaterials that resemble SOD have been created over time. Approximately 100 different types of nanozymes have been discovered to have SOD-like activity thus far, the majority of which are composed of components including carbon, nitrogen, oxygen, sulfur, and transition metals like iron (Fe) and cobalt (Co) [285]. A new class of fluorescent nanoparticles called carbon dots (or CDs) has drawn interest recently because to their biocompatibility and adaptability for use in cancer treatment and diagnostic (theragnostic) applications. Although their ability to treat a wide range of illnesses is likely to have a significant impact, the majority of the research now focuses on their use as cancer treatments. There are numerous CD formats, each with unique qualities appropriate for a range of uses. Graphitic carbon dots, graphitic carbon nitride dots, carbon black dots, amorphous carbon quantum dots (CQDs), polymeric dots, polymer/carbon hybrid dots, and co-doped (heteroatom) CDs are a few examples of these dots [286]. By changing the drug/dot constructions’ polarity, total charge, and solubility, they can help medications move through the body to areas where they could not go by themselves (i.e., transportation of drugs across the blood–brain barrier) [286,287].

Long-term inflammatory remission was reported to be anticipated with C-dots nanozyme, a new therapeutic strategy for IBD with negligible systemic toxicity [288]. In 2023, a report showed that hepatic ischemia–reperfusion injury can be managed with carbon dot nanozymes as free radical scavengers by controlling the liver inflammatory network and preventing apoptosis [289]. By scavenging ROS, the C-dots demonstrated to have greater anti-inflammatory effects in the DSS-induced colitis mice compared to 5-ASA, the clinical first-line treatment medication [290].

The capacity of nanozymes with superoxide dismutase (SOD)-like activity to scavenge superoxide anion, the source of the majority of reactive oxygen species in vivo, has garnered growing attention. SOD nanozymes have not yet been able to match the activity of real enzymes, though. With a catalytic activity of over 10,000 U/mg, we present a carbon dot (C-dot) SOD nanozyme that is on par with real enzymes. We demonstrate by specific chemical changes and theoretical computations that the SOD-like activity of C-dots depends on the carbonyl groups conjugated with the π-system for electron transfer and the hydroxyl and carboxyl groups for binding superoxide anions. Additionally, C-dot SOD nanozymes have the intrinsic ability to target oxidation-damaged cells and successfully shield neuron cells during an ischemic stroke model of male mice [291]. It has been reported that a new carbon dot (C-dot) superoxide dismutase (SOD) nanozyme with red fluorescence at 683 nm and high SOD-like activity of >4000 U mg^−1^ has great potential for imaging the nanozyme’s biodistribution in vivo and treating acute lung injury [292]. Additionally, synthetic SOD mimetics are under investigation to replicate or improve the enzyme’s antioxidant activity while minimizing toxicity and immune responses. With continued research into optimizing SOD’s activity and targeting, it may become an effective tool for managing oxidative stress-related conditions, including neurodegenerative diseases, ischemia–reperfusion injuries, and cancer [293].

In summary, carbon-dot based nanoenzymes with SOD-like activity offer a promising therapeutic strategy for managing oxidative stress-related diseases due to their high catalytic efficiency, biocompatibility, and disease targeting abilities. Continued research into optimizing their structure and function may enable their clinical translation for conditions such as inflammation, ischemia, and neurodegenerative diseases, etc.

## 11. Synthetic SOD Mimetics

Synthetic SOD mimetics are designed to replicate the SOD’s antioxidant function, and to overcome some limitations of natural SODs, such as poor bioavailability. However, the efficacy of these mimetics in replicating the complex catalytic activities of SOD in vivo remains variable. SOD mimetics have better pharmacokinetic properties, pharmacodynamic variability, and reduced antigenicity. Some mimetics lack the efficiency and specificity required to compete with native SOD enzymes, which can diminish their therapeutic potential [62]. The production of O_2_^•−^ is common to both radiation therapy and chemotherapy. Fortunately, ROS generation after radiation and chemotherapy is typically higher in cancer cells than in normal tissues due to metabolic differences between cancer and normal cell metabolism as well as improved targeting approaches. Nonetheless, the quantities of ROS produced in healthy tissues have the potential to cause serious damage. Mapuskar and colleagues have presented a beautiful review summarizing the studies regarding the utilization of superoxide dismutase mimetics to enhance radiation therapy response while protecting normal tissues [294]. The therapy of neurological disorders makes considerable use of SOD mimetics such as Metalloporphyrin, Mn (II)-cyclic polyamines, Nitroxides, and Mn (III)–Salen complexes. Future treatments for a variety of diseases with oxidative stress-mediated pathology appear to be promising when it comes to SODs and SOD mimetics [213]. Although SOD mimetics are less likely to be degraded by proteases, they can pose toxicity risks at high concentrations. An over-reduction in ROS can lead to excessive suppression of important cellular processes, potentially causing harm to the patient [182].

Thus, synthetic SOD mimetics offer a promising alternative to natural SOD enzymes by improving stability, bioavailability, and targeting. While they show potential in enhancing therapeutic outcomes in cancer and other oxidative stress-related pathologies, challenges still remain like variable efficacy, lack of the standardization of doses, toxicity linked with high doses, etc.

## 12. Administration of SOD

SOD supplementation may be a useful preventive measure to lower the risk of excessive formation of free radicals. Nevertheless, SOD’s quick clearance compromises its effectiveness. In recent decades, several strategies to increase SOD’s bioavailability have been investigated [62]. In irradiated rodents, subcutaneous injection of bovine Cu/Zn-SOD inhibits collagen deposition, which is the cause of the fibrosis [295]. 

In irradiated pigs, Lefaix and colleagues found that fibrotic regions are reduced after three weeks of curative SODs treatment (intramuscular injections) [296]. Lastly, topical treatment of tomato Cu,Zn-SOD [297] or intramuscular injection of bovine Cu,Zn-SOD [298] near the injured areas has been proven in clinical investigations to reduce radiotherapy-induced fibrotic areas for a number of days. Many investigations have reported the positive effects of SOD administration through various routes including Intravenous injection of human Mn-SOD [299], oral administration [300,301], retrograde intracoronary administration [302], intragastric administration [303], intraperitoneal administration [304], subcutaneous administration [279], intravenous administration [305], intracerebroventricular administration [306], etc.

## 13. Conclusions

Superoxide dismutase (SOD) has been shown in clinical and preclinical trials to have therapeutic potential against oxidative stress and oxidative stress-induced disease in a number of scientific studies conducted in recent years. There is a strong correlation with kinase, which makes it necessary to explore new target sites and introduce novel formulation techniques like gene modulation, nano-formulations, and click chemistry. Numerous studies in this review have established SOD’s physiological significance and therapeutic potential. SODs have a lot of potential in the medical field because of their antioxidative properties. The results of studies conducted on humans and animals that demonstrate the benefits of SOD enzyme modulation in lowering the development of oxidative stress in a variety of methods are covered in this review. In conclusion, while superoxide dismutases (SODs) show great therapeutic potential in combating oxidative stress-related diseases, challenges such as poor bioavailability, limited specificity, and immune responses hinder their clinical application. Future advancements in delivery systems, synthetic mimetics, and targeted therapies could overcome these limitations, paving the way for more effective use of SOD in treating conditions like neurodegenerative diseases, cancer, and ischemia.

## Figures and Tables

**Table 1:** A summary of different studies emphasizing the implications of SOD in various diseases.

Disease	Type of SOD	Subjects	Conclusion of the Study	Reference
Breast cancer	Cu/Zn-SOD and Mn-SOD	70	Patients with breast cancer may have elevated SOD expression in response to the buildup of free radicals. The improvement in the expression of activated SODs may be attributed to anastrozole treatment.	[63]
Breast cancer	SOD2	80	A poor prognosis was associated with increased SOD2 expression.	[64]
Glioblastomas	Mn-SOD	30	Using this SOD protein as a marker could aid in developing treatment plans for glioblastoma patients.	[65]
Glioblastomas	SOD	100	Following treatment, SOD and CAT levels increased and the hydrogen inhalation group’s overall impact was noticeably superior to that of the conventional group.	[66]
Colorectal cancer	Serum SOD	176	The total cohort’s risk of colorectal cancer (CRC) decreased as SOD increased, with the lowest risk being seen in the fourth quartile of SOD levels relative to the first.	[67]
Pancreatic cancer	SOD1	50	In pancreatic cancer and chronic pancreatitis, oxidative stress indicators were elevated and antioxidant defense system capacity was decreased.	[68]
Gastric cancer	Serum SOD	34	A poor prognosis for individuals with far-advanced (stage IV) stomach cancer may come from a decrease in serum SOD activity in older patients, which could be caused by a weakened host antioxidant defense.	[69]
Oral cancer	NA	87	The amount of SOD falls and the amount of NO activity rises during carcinogenesis and tumor growth. These NO and SOD levels may also be used as prognostic indicators and treatment goals for patients with this type of illness.	[70]
Lung cancer	Cu-Zn SOD	32	Patients with NSCLC and SCLC had overall higher levels of NOradical dot, MDA, and TGHS as well as activity of XO, CAT, CuZn SOD, and unaltered GSH-Px GST as compared to the control group.	[71]
Brain cancer (Intracranial neoplasm)	Erythrocyte SOD	30	Significant oxidative stress in brain tumors may be triggered by a considerable decline in antioxidant levels. Furthermore, the degree of aggressiveness in brain tumors may be indicated by the drop in antioxidant levels.	[72]
Brain Tumor	Total SOD (Both Mn-SOD and Cu,Zn-SOD)	32	CAT activity was 106.3% greater and SOD activity was much lower in brain tumor tissue compared to controls.	[73]
Inflammatory disease (Periprosthetic joint infection)	Serum SOD	50	Serum SOD demonstrated significant promise in periprosthetic joint infection diagnosis.	[74]
HEV-induced liver failure	Serum SOD	30	SOD levels were higher in patients with HEV-induced liver failure than in HEV-AVH patients and healthy controls.	[75]
Cardiovascular disease	SOD	50	Patients with ST elevated myocardial infarction (STEMI) and non-ST elevated myocardial infarction (NSTEMI) had considerably lower levels of SOD and catalase, which may indicate that they are experiencing elevated oxidative stress.	[76]
Coronary artery disease	SOD2	150	One marker gene for CAD susceptibility is the SOD2 locus.	[77]
Aging	SOD3	1100	EC-SOD has positive effects on diabetes mellitus in the elderly and works in tandem with adiponectin.	[78]
Aging	Plasma SOD	78	Smoking and low superoxide dismutase (SOD) levels are risk factors for early aging in women between the ages of 20 and 35.	[79]
Rheumatoid arthritis	Cu,Zn-SOD	28	The study documented a negative correlation between CRP levels and Cu,Zn-SOD activities.	[80]
Rheumatoid arthritis	SOD	54	Serum albumin levels and SOD concentrations in the peripheral antioxidant status were better after multigrain supplementation than in the control group.	[81]
Amyotrophic lateral sclerosis (ALS)	SOD	24	The cerebrospinal fluid SOD and NO level might serve as useful biomarkers for functional disorder and progression of the disease.	[82]
Huntington’s disease	SOD	375	Triglycerides, high-density lipoproteins, low-density lipoproteins, cholesterol, and blood SOD did not significantly differ between patients and controls.	[83]
Parkinson’s disease	Erythrocyte SOD	29	There may be distinct clinical subgroups of Parkinson’s disease (PD) that can be distinguished by a biological marker, as suggested by the variation in SOD activity in clinically diverse subgroups.	[84]
Alzheimer’s disease	Cu,Zn-SOD	44	Compared to the controls, the patients’ red blood cells had substantially less SOD activity.	[85]

## Data Availability

No new data were created or analyzed in this study.
